# The I7L protein of African swine fever virus is involved in viral pathogenicity by antagonizing the IFN-*γ*-triggered JAK-STAT signaling pathway through inhibiting the phosphorylation of STAT1

**DOI:** 10.1371/journal.ppat.1012576

**Published:** 2024-09-26

**Authors:** Meilin Li, Xinyuan Liu, Dingkun Peng, Meng Yao, Tao Wang, Yijing Wang, Hongwei Cao, Yanjin Wang, Jingwen Dai, Rui Luo, Hao Deng, Jiaqi Li, Yuzi Luo, Yongfeng Li, Yuan Sun, Su Li, Hua-Ji Qiu, Lian-Feng Li

**Affiliations:** State Key Laboratory for Animal Disease Control and Prevention, National African Swine Fever Para-Reference Laboratory, National High-Containment Facilities for Animal Disease Control and Prevention, Harbin Veterinary Research Institute, Chinese Academy of Agricultural Sciences, Harbin, China; Pirbright Institute, UNITED KINGDOM OF GREAT BRITAIN AND NORTHERN IRELAND

## Abstract

Cell-passage-adapted strains of African swine fever virus (ASFV) typically exhibit substantial genomic alterations and attenuated virulence in pigs. We have indicated that the human embryonic kidney (HEK293T) cells-adapted ASFV strain underwent genetic alterations and the *I7L* gene in the right variable region was deleted compared with the ASFV HLJ/2018 strain (ASFV-WT). A recent study has revealed that the deletion of the *I7L*-*I11L* genes results in attenuation of virulent ASFV *in vivo*, but the underlying mechanism remains largely unknown. Therefore, we hypothesized that the deletion of the *I7L* gene may be related to the pathogenicity of ASFV in pigs. We generated the *I7L* gene-deleted ASFV mutant (ASFV-ΔI7L) and found that the *I7L* gene deletion does not influence the replication of ASFV in primary porcine alveolar macrophages (PAMs). Using transcriptome sequencing analysis, we identified that the differentially expressed genes in the PAMs infected with ASFV-ΔI7L were mainly involved in antiviral immune responses induced by interferon gamma (IFN-*γ*) compared with those in the ASFV-WT-infected PAMs. Meanwhile, we further confirmed that the I7L protein (pI7L) suppressed the IFN-*γ*-triggered JAK-STAT signaling pathway. Mechanistically, pI7L interacts with STAT1 and inhibits its phosphorylation and homodimerization, which depends on the tyrosine at position 98 (Y98) of pI7L, thereby preventing the nuclear translocation of STAT1 and leading to the decreased production of IFN-*γ*-stimulated genes. Importantly, ASFV-ΔI7L exhibited reduced replication and virulence compared with ASFV-WT in pigs, likely due to the increased production of IFN-*γ*-stimulated genes, indicating that pI7L is involved in the virulence of ASFV. Taken together, our findings demonstrate that pI7L is associated with pathogenicity and antagonizes the IFN-*γ*-triggered JAK-STAT signaling pathway via inhibiting the phosphorylation and homodimerization of STAT1 depending on the Y98 residue of pI7L and the Src homology 2 domain of STAT1, which provides more information for understanding the immunoevasion strategies and designing the live attenuated vaccines against ASFV infection.

## Author summary

Deletion of pivotal virulence-associated genes to develop live attenuated African swine fever (ASF) vaccines is considered as a promising strategy. Our previous research demonstrated that the *I7L* gene of the human embryonic kidney (HEK293T) cells-adapted African swine fever virus (ASFV) strain was completely lost, and a recent study has revealed that the deletion of the *I7L*-*I11L* genes resulted in ASFV attenuation, suggesting that the deletion of the *I7L* gene seemed to be related to the pathogenicity of ASFV in pigs. In this study, we investigated the function of the I7L protein (pI7L) in the ASFV replication cycle *in vitro* as well as evaluated the pathogenicity of the *I7L* gene-deleted ASFV mutant *in vivo*. Our results showed that the deletion of *I7L* gene does not influence the replication of ASFV in primary porcine alveolar macrophages. However, pI7L is involved in the virulence of ASFV, and directly interacts with STAT1 and antagonizes the IFN-*γ*-triggered JAK-STAT signaling pathway via inhibiting the phosphorylation and homodimerization of STAT1 depending on the tyrosine at position 98 of pI7L and the Src homology 2 domain of STAT1. This study not only facilitates the understanding of ASFV immunoevasion strategies, but also provides a novel target for the development of live attenuated vaccines against ASF.

## Introduction

African swine fever (ASF), caused by African swine fever virus (ASFV), is a highly contagious infectious disease of domestic pigs and wild boars with a mortality rate approaching 100% for the peracute form [1]. ASF has been epidemic in Africa and Europe for over a century [2]. In 2018, it spread to China and other Asian countries, causing significant losses to the pig industry [3]. Currently, only two live attenuated vaccines (LAVs) against ASF have been licensed for restricted vaccination in Vietnam [4]. Disease surveillance and strict biosecurity measures continue to be the primary strategies for preventing and controlling ASF [5].

ASFV is mainly propagated in primary porcine monocytes and macrophages. Given the limited number of appropriate cell lines capable of supporting ASFV replication, the basic research and vaccine development of ASFV are severely hindered [6]. Recently, several established cell lines, such as IPAMs (immortalized porcine alveolar macrophages), COS-1 (African green monkey kidney cells), WSL (wild boar lung cell line), MA-104 (African green monkey kidney epithelial cells), ZMAC-4 (Zuckermann macrophage-4), and IPKMs (immortalized porcine kidney-derived macrophages), have been utilized to propagate and titrate limited strains of ASFV [7–10]. Furthermore, it is useful to establish a cell adapted ASFV strain from the highly virulent parent strain, such as the virulent strain BA71V, to elucidate viral gene functions, virulence-associated genes, and immunoevasion mechanisms [11]. In our previous study, the human embryonic kidney (HEK293T) cells-adapted ASFV HLJ/2018-P61 (ASFV-P61) strain was obtained from the highly virulent ASFV HLJ/2018 (ASFV-WT) strain, which was isolated from the spleen of an ASFV-infected pig in Heilongjiang, China, in 2018, by adaptive passaging in HEK293T cells [12]. During the adaptation of ASFV *in vitro*, the increasing number of passages in these cell lines led to observable genotypic and phenotypic changes, accompanied by associated genome mutations.

Whole genome sequencing was conducted to identify the mutations between ASFV-WT and ASFV-P61 strains. A large number of deletions and mutations were detected in the variable regions, with the *I7L* gene in the right variable region exhibiting twelve nucleotide mutations. One of the pivotal mutations is the initiation codon (ATG to ATA), resulting in complete loss of the *I7L* gene translation. The *I7L* gene also exhibited a similar deficiency in Vero cells-adapted strains, such as the ASFV Georgia2010 strain (ASFV-G)/V30/60 and BA71V [13]. Several passage-adapted ASFV strains typically exhibit significant genomic alterations and decreased virulence in pigs, such as L60V (Vero cells-adapted L60), E70Ms14 (MS monkey kidney cells-adapted E70), and E75CV1 (CV1 cells-adapted E75) [13–15]. The deletion of the *I7L* gene in various cells-adapted ASFV strains suggests its potential involvement in ASFV adaptation, leading to the hypothesis that the absence of the *I7L* gene contributes to ASFV attenuation in pigs.

Although the deletion of virulence-associated genes has been utilized in the development of ASF LAVs, the mechanisms underlying the attenuation of some vaccine candidates remain poorly understood. The ASFV SY18ΔI7L-I11L mutant, characterized by the deletion of the *I7L-I11L* genes from its parental strain ASFV SY18, has been constructed and its biological properties analyzed [16]. The results show that the growth kinetics of SY18ΔI7L-I11L are similar to those of the ASFV SY18 *in vitro*; however, SY18ΔI7L-I11L exhibits attenuated pathogenicity and confers complete protection against the challenge with virulent ASFV SY18 *in vivo*, indicating that one or more genes in *I7L*-*I11L* may play a critical role in attenuating virulence. Interestingly, SY18ΔI7L-I11L induced a higher level of interferon gamma (IFN-*γ*) than did the ASFV SY18 strain, revealing that the *I7L*-*I11L* genes may contribute to IFN-*γ* production and IFN-*γ*-mediated antiviral response. Hence, further investigation is necessary to elucidate the functions and attenuation mechanisms of these individual genes.

The *I7L-I11L* gene cluster, comprising the *I7L*, *I8L*, *I9R*, *I10L*, and *I11L* genes, are located in the right variable region of the ASFV genome. The *I7L* gene, a member of *MGF100* [17], resides on the negative strand between nucleotides (nt) 180726 and 181034 in the ASFV-WT genome and encodes the I7L protein (pI7L) consisting of 102 amino acid (aa) residues [18]. The data showed that the aa 9–91 of pI7L form a Src homology 2 (SH2) domain (https://www.ebi.ac.uk/interpro/protein/UniProt/A0A0C5B0D0/). However, as yet, the crystal structure of pI7L remains unresolved. The proteins that contain the SH2 domain, comprising approximately 100 aa residues, play important roles in the regulation of intracellular signal transduction [19,20]. The SH2 domain specifically recognizes the phosphorylated tyrosine (pTyr)-containing ligand proteins, a process that is highly dependent on the specific aa sequences directly C-terminal to the pTyr [21,22]. Despite the potential presence of an SH2 domain in pI7L, its functional roles remain unclear.

IFN-*γ* coordinates a diverse array of cellular processes by regulating the transcription of immunologically relevant genes [23], serving as a critical cytokine in the immune response against viral and intracellular bacterial infections [24,25]. Upon stimulation with IFN-*γ*, it binds to both the IFN-*γ* receptor 1 (IFNGR1) and IFNGR2, promoting the proximity of Janus kinase 1 (JAK1) and JAK2 for their phosphorylation, along with that of IFNGR1 [26]. The cooperative activation of JAK1 and JAK2 results in the phosphorylation of signal transducers and activators of transcription 1 (STAT1) at tyrosine position 701 (Y701) [27,28], leading to the formation of STAT1 homodimers that translocate to the nucleus to induce transcription of various genes involved in immune responses and cell proliferation [29]. The C-X-C motif chemokines (CXCLs), such as CXCL9 and CXCL10, are secreted by various cells in response to IFN-*γ* and serve as chemotactic attractants for immune cells [30]. It has been shown that the ASFV-encoded protein MGF505-7R antagonizes the JAK-STAT1 signaling pathway by impairing the IFN-*γ*-mediated activation of JAK1 and JAK2 [25]. However, the mechanisms by which other ASFV-encoded proteins modulate the JAK-STAT1 signaling to evade the immune response remain elusive.

In this study, we demonstrated that the *I7L* gene is dispensable for ASFV replication in primary porcine alveolar macrophages (PAMs). In addition, pI7L has been identified as a virulence factor of ASFV, directly interacting with STAT1 and exerting a negative regulatory effect on the IFN-*γ*-triggered JAK-STAT signaling pathway by suppressing the phosphorylation and homodimerization of STAT1. This inhibition is contingent upon the tyrosine residue at position 98 (Y98) of pI7L and the SH2 domain of STAT1. Our findings offer novel insights into the immunoevasion strategies employed by ASFV.

## Results

### Conservation of pI7L among different ASFV isolates and transcription of the *I7L* gene in the ASFV replication cycle

Cell-passage-adapted ASFV strains typically exhibit substantial genomic alterations and attenuated phenotype in pigs. To identify which genes are involved in the virulence of ASFV, we analyzed the genetic alterations of ASFV during serial passaging in HEK293T cells. The consensus genomic sequences from 12 passages were assembled and compared with the genome of ASFV-WT [12]. The sequence analysis revealed twelve nucleotide mutations within the 3′-terminus of the *I7L* gene, including a critical mutation (ATG to ATA) at the initiation codon that abolishes the translation of the *I7L* gene (S1A Fig). Notably, a similar deletion has been reported in Vero cells-adapted strains, like ASFV-G/V30/60 and BA71V (S1B Fig), implying a potential association between the *I7L* gene and the pathogenicity of ASFV.

**Fig 1:**
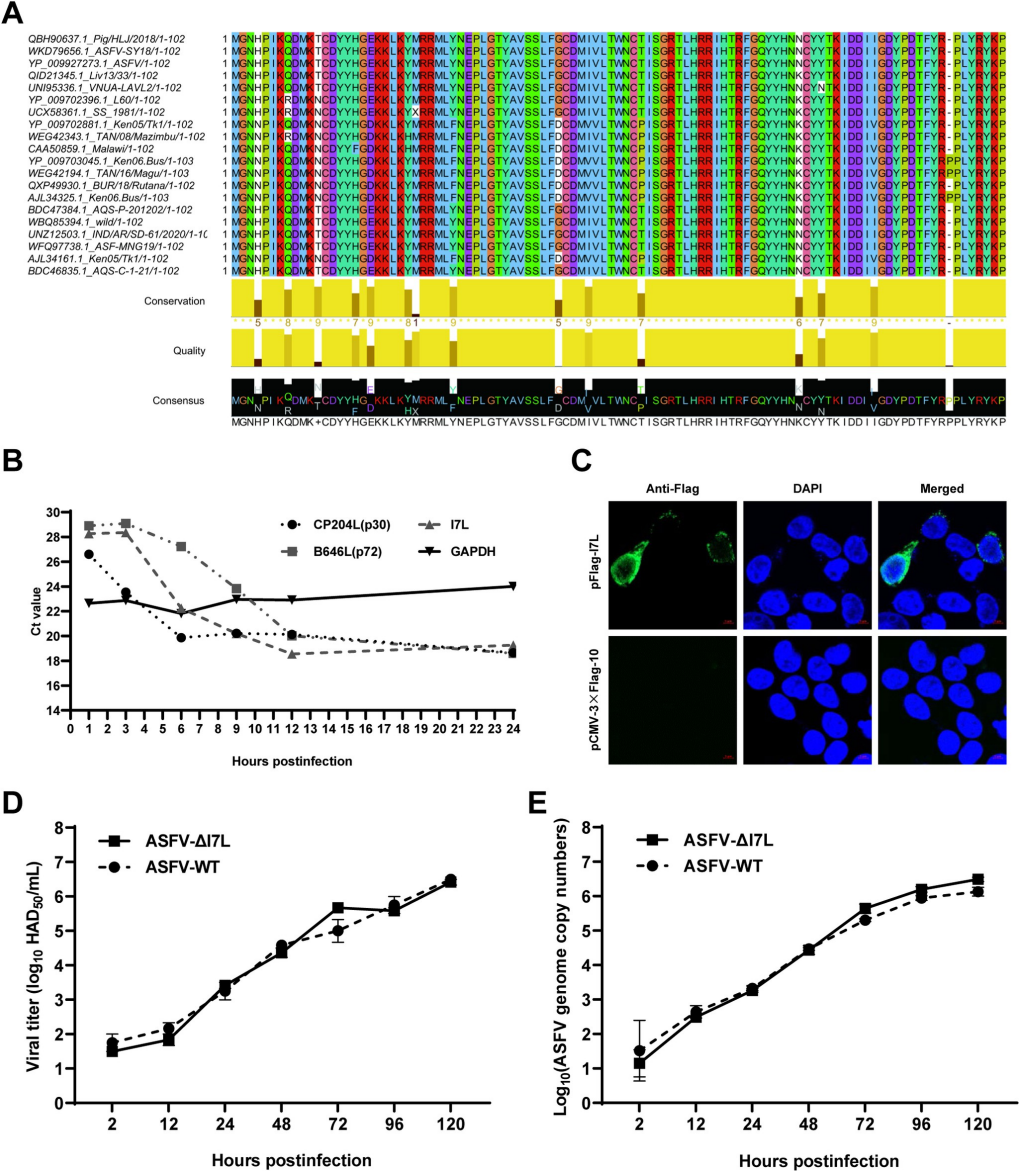
The biological characteristics of the I7L protein (pI7L). (A) Multiple sequence alignment of pI7L among 20 ASFV strains. The homology analysis of pI7L was conducted using the Jalview software version 2.11.1.4. (B) The transcription dynamics of the *I7L* gene. Primary porcine alveolar macrophages (PAMs) were infected with the ASFV HLJ/2018 strain (ASFV-WT) at a multiplicity of infection of 1. At 1, 3, 6, 9, 12, and 24 hours postinfection (hpi), the average cycle threshold (CT) values of the PAMs were examined by a reverse transcription-quantitative PCR (RT-qPCR) using the primers targeting *I7L*, *B646L* (p72), *CP204L* (p30), and *GAPDH*. (C) Intracellular localization of pI7L. HEK293T cells were transfected with pFlag-I7L (0.5 μg). At 24 hpi, the cells were fixed and stained with a mouse anti-Flag monoclonal antibody and the nuclear marker DAPI and then examined by laser confocal microscopy. Bars, 5 μm. (D and E) The replication kinetics of ASFV-ΔI7L in PAMs. PAMs were seeded into 24-well plates and infected with ASFV-ΔI7L or ASFV-WT at a multiplicity of infection (MOI) of 0.01. At the indicated time points, the viral titers (D) and the genome copies (E) were determined by a quantitative real-time PCR (qPCR) and hemadsorption assay, respectively. Error bars denote the standard errors of the means. All the data were analyzed using the one-way ANOVA. ***, *P* < 0.001; ns, not significant.

The *I7L* gene of ASFV-WT is located in the reverse strand between nt 180726 and 181034 in the viral genome, encoding the pI7L consisting of 102 aa. The aa sequences of pI7L from 20 ASFV isolates were compared by multiple sequence alignment using the Jalview software version 2.11.1.4. The data showed a very high degree of aa sequence similarity, ranging from 89.2% to 100.0% identity among isolates containing the same or different forms of pI7L (Fig 1A), indicating that pI7L is highly conserved within these isolates. The transcription kinetics of the *I7L* gene was assessed using a reverse transcription-quantitative PCR (RT-qPCR). Compared with *CP204L* (p30) that was included as an early-expressed viral gene, and *B646L* (p72) that was included as a late-expressed viral gene, the *I7L* gene transcription was decreased at 3 hours postinfection (hpi), but rapidly increased between 3 and 6 hpi, following a pattern similar to the early-transcribed gene *CP240L* (Fig 1B). Consistent with a previous study [31], our data also demonstrated that the *I7L* gene is an early transcription gene of ASFV. Furthermore, we confirmed that pI7L was localized in the cytoplasm using laser confocal microscopy (Fig 1C).

### The *I7L* gene deletion does not influence the replication of ASFV in PAMs

To investigate the roles of pI7L in ASFV infection in PAMs, ASFV-ΔI7L, an ASFV mutant lacking the *I7L* gene (S2A Fig), was generated based on DNA homologous recombination as described previously [32]. ASFV-ΔI7L was purified after nine rounds of limiting dilution purification based on enhanced green fluorescent protein (EGFP) screening (S2B Fig). The recombinant virus ASFV-ΔI7L was subjected to PCR targeting the *I7L* gene (left) and *p72EGFP* cassette sequence (right), excluding parental ASFV contamination (S2C Fig). Normal viral particles (white arrows) were detected in the ASFV-ΔI7L-infected PAMs by transmission electron microscopy (TEM), similar to the mature virions observed in the ASFV-WT-infected PAMs (S2D Fig). The "rosettes" of red blood cells were formed in both the ASFV-ΔI7L- or ASFV-WT-infected PAMs by hemadsorption assay, indicating that the deletion of the *I7L* gene does not alter the hemadsorption characteristic of ASFV (S2E Fig). Furthermore, a comprehensive analysis of the whole genome of ASFV-ΔI7L was conducted using next-generation sequencing (NGS). Compared with ASFV-WT, no undesired mutations were detected in the genome of ASFV-ΔI7L, except for a 309-bp deletion of the *I7L* gene replaced by the p72-EGFP expression cassette (S2F Fig). We subsequently analyzed the growth kinetics of ASFV-ΔI7L *in vitro*. The growth profile of ASFV-ΔI7L was similar to that of ASFV-WT in PAMs within 120 hpi (Fig 1D and 1E), indicating that the deletion of the *I7L* gene does not affect the replication of ASFV in PAMs.

### Differentially expressed genes (DEGs) in the ASFV-ΔI7L-infected PAMs by RNA sequencing (RNA-seq) analysis

**Fig 2:**
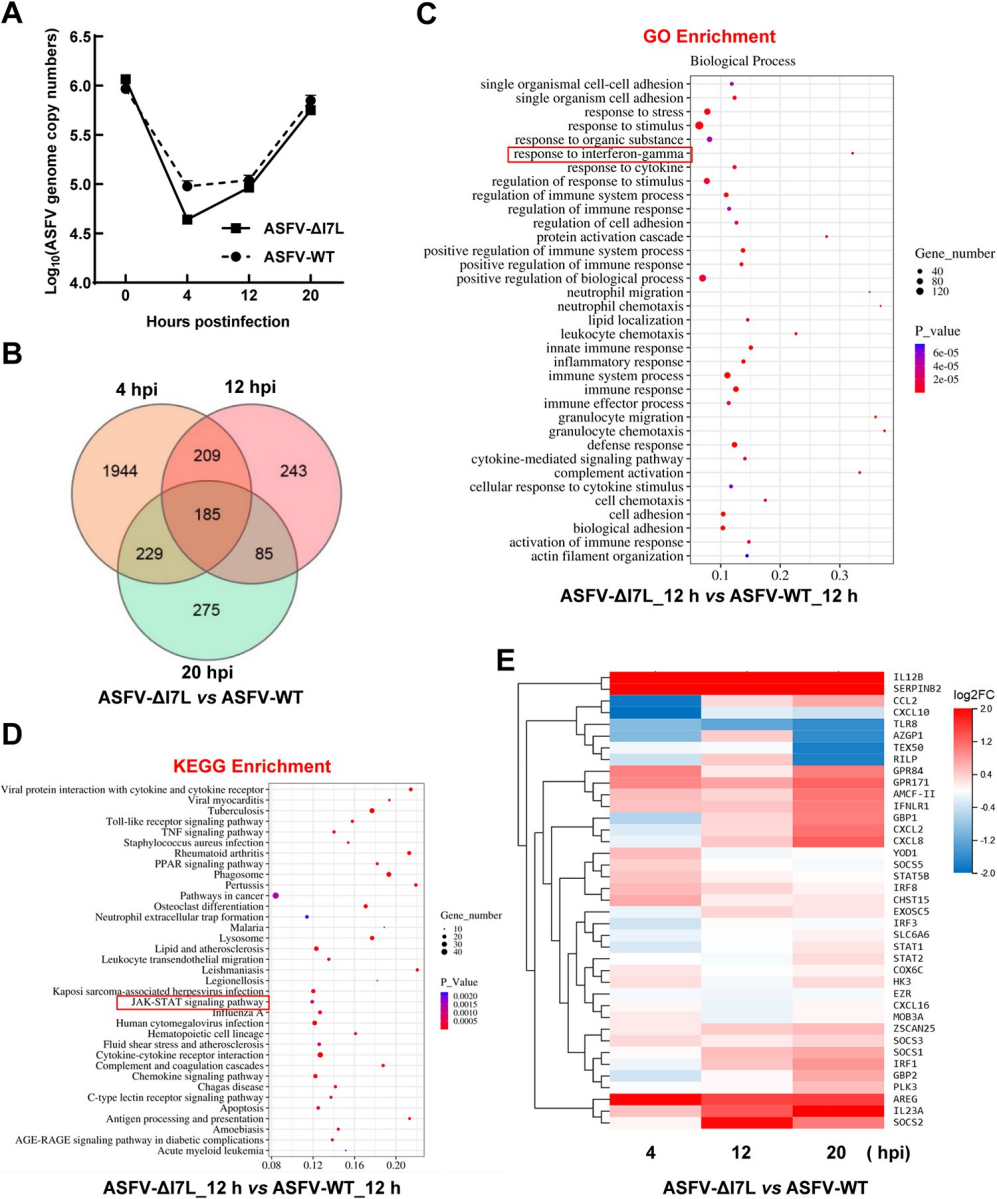
Differential expression profiling in the ASFV-ΔI7L-infected primary porcine alveolar macrophages (PAMs) by RNA sequencing analysis. (A) Identification of ASFV infection. PAMs were infected with ASFV-ΔI7L or ASFV-WT at a multiplicity of infection (MOI) of 1. The ASFV genome copies were determined by a quantitative real-time PCR (qPCR) at 4, 12, and 20 hours postinfection (hpi). (B) Venn diagrams of the differentially expressed genes (DEGs) in the ASFV-ΔI7L- versus (*vs*.) ASFV-WT-infected PAMs. (C and D) The bioinformatics analysis of DEGs. The gene ontology (GO) enrichment (C) and the Kyoto Encyclopedia of Genes and Genomes (KEGG) enrichment (D) analyses were performed in the ASFV-ΔI7L- *vs*. ASFV-WT-infected PAMs at 12 hpi. (E) Heat map of the DEGs induced by ASFV-ΔI7L *vs*. ASFV-WT at 4, 12, and 20 hpi. Error bars denote the standard errors of the means. All the data were analyzed using the one-way ANOVA. ***, *P* < 0.001; ns, not significant.

To investigate the roles of pI7L in ASFV replication, the DEGs of the PAMs infected with ASFV-ΔI7L or ASFV-WT at a multiplicity of infection (MOI) of 1 were analyzed by RNA-seq analysis at 4, 12, and 20 hpi (Fig 2A). A total of 18 samples were analyzed, resulting in 43.87–44.52 M clean reads. Alignment with the reference genome showed a high alignment rate, ranging from 91.07% to 93.85%. Compared with the ASFV-WT-infected PAMs, ASFV-ΔI7L infection of PAMs resulted in differential expression of 2567, 722, and 774 genes at 4, 12, and 20 hpi, respectively. Additionally, we employed a Venn diagram to illustrate the number of genes that exhibited changes at 4, 12, and 20 hpi. The results showed that only 185 genes exhibited consistent changes across all three time points (Fig 2B). The DEGs were subjected to gene ontology (GO) enrichment analysis at 4 (S3A Fig), 12 (Fig 2C), and 20 (S3C Fig) hpi, and to Kyoto Encyclopedia of Genes and Genomes (KEGG) enrichment analysis at 4 (S3B Fig), 12 (Fig 2D), and 20 (S3D Fig) hpi. Compared with the ASFV-WT-infected PAMs, the DGEs in the ASFV-ΔI7L-infected PAMs showed a significant enrichment in cellular response related to the IFN-*γ* and JAK-STAT signaling cascades (red rectangle) at 12 and 20 hpi. Importantly, the heat map of the DEGs showed that ASFV-ΔI7L induced higher production of IFN-*γ*-stimulated genes (ISGs), such as *IRF1*, *GBP1*, *SOCS1*, and *CXCL10*, than did ASFV-WT in PAMs at 12 and 20 hpi (Fig 2E). Collectively, these data suggest that the function of pI7L is involved in the IFN-*γ-*triggered JAK-STAT signaling pathway.

### The *I7L* gene-deleted ASFV mutant activates the IFN-*γ*-triggered JAK-STAT signaling pathway

**Fig 3:**
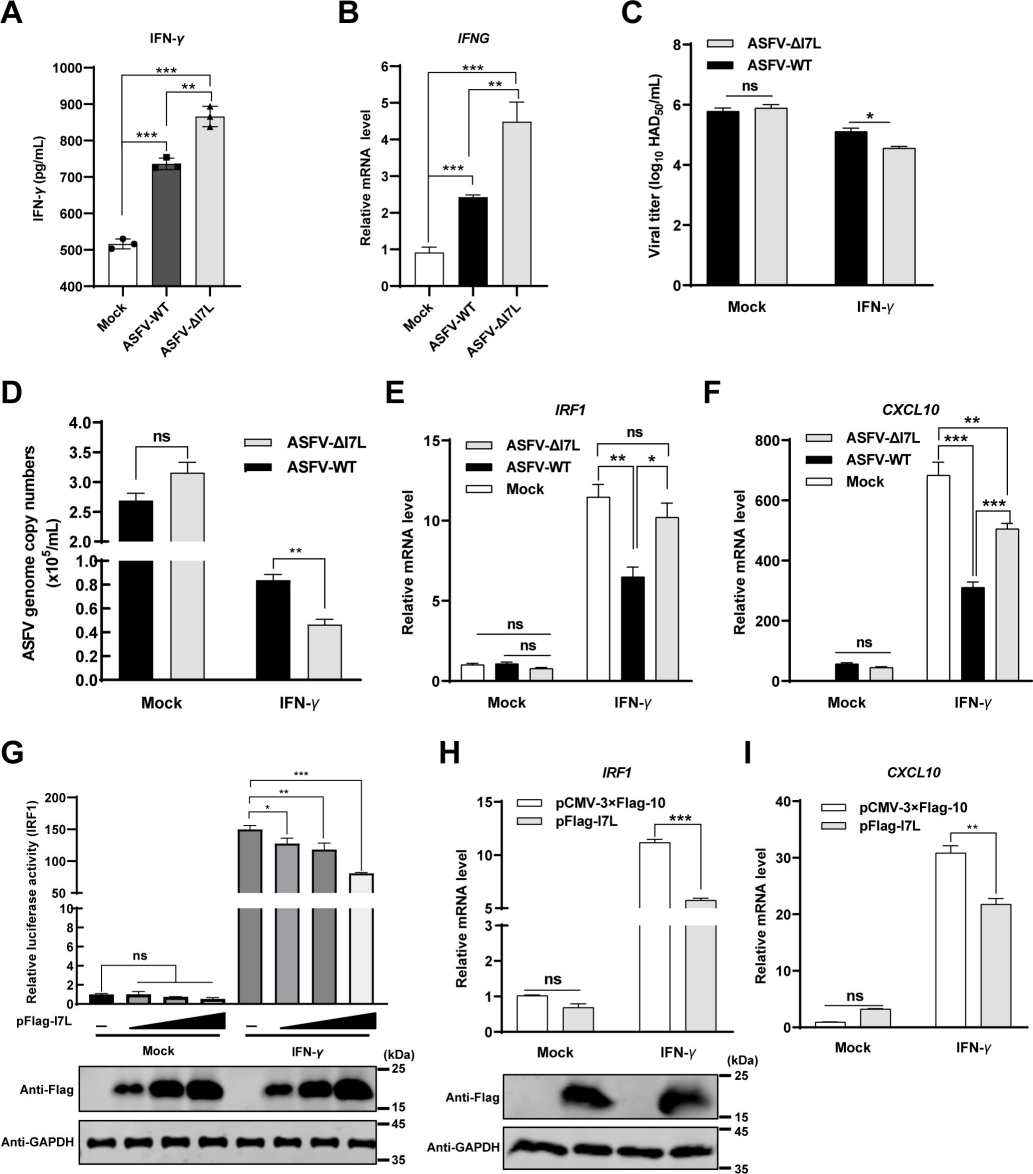
pI7L suppresses the IFN-*γ*-triggered JAK-STAT signaling pathway. (A and B) ASFV-ΔI7L induces higher IFN-*γ* production than does ASFV-WT. Primary porcine alveolar macrophages (PAMs) were either mock-infected or infected with ASFV-ΔI7L or ASFV-WT at a multiplicity of infection (MOI) of 1. At 20 hours postinfection (hpi), the translational level of IFN-*γ* (A) and the transcriptional level of *IFNG* (B) were examined using the ELISA kit and reverse transcription-quantitative PCR (RT-qPCR), respectively. (C and D) pI7L antagonizes the antiviral activity of IFN-*γ*. PAMs were treated with IFN-*γ* or mock-treated for 24 hours, followed by infection with ASFV-ΔI7L or ASFV-WT at an MOI of 0.1 for 48 hours. The viral titers (C) and the genome copies (D) were determined by hemadsorption assay and quantitative real-time PCR (qPCR), respectively. (E and F) ASFV-ΔI7L induces higher IFN-*γ*-stimulated genes (ISGs) production than does ASFV-WT. PAMs were mock-infected or infected with ASFV-ΔI7L or ASFV-WT at an MOI of 1. At 24 hpi, the transcriptional levels of *IRF1* (E) and *CXCL10* (F) in the cell lysates were quantified by RT-qPCR. (G) pI7L inhibits the activation of the *IRF1* promoter in a dose-dependent manner. HEK293T cells grown in 24-well plates were transfected with pFlag-I7L (0.1, 0.2, and 0.5 μg) together with the promoter reporter plasmid pIRF1-Fluc (100 ng) and the internal reference reporter plasmid pSV40-Rluc (10 ng). At 24 hours posttransfection (hpt), the transfected cells were mock-treated or treated with IFN-*γ* (20 ng/ml) for another 12 hours. The cells were lysed and the reporter activity was analyzed with a dual-luciferase assay kit. (H and I) pI7L inhibits the production of ISGs. HEK293T cells were transfected with pFlag-I7L or p3xFlag-CMV-10. At 24 hpt, the cells were mock-treated or treated with IFN-*γ* (20 ng/ml) for another 12 hours, and then the total RNA was extracted, and the transcriptional levels of *IRF1* (H) and *CXCL10* (I) in the cell lysates were quantified by a relative RT-qPCR. Error bars denote the standard errors of the means. All the data were analyzed using the one-way ANOVA. ***, *P* < 0.001; ns, not significant.

To determine whether pI7L is involved in the IFN-*γ* production and IFN-*γ*-triggered signaling pathway, the IFN-*γ* production were examined. The results demonstrated that both ASFV-ΔI7L and ASFV-WT significantly enhanced the protein expression of IFN-*γ* (Fig 3A) as well as the transcriptional level of the *IFNG* gene (Fig 3B), but ASFV-ΔI7L induced higher expression of IFN-*γ* than did ASFV-WT, indicating that pI7L antagonizes the production of IFN-*γ*. Furthermore, PAMs were treated with IFN-*γ* or mock-treated for 24 hours, followed by infection with ASFV-ΔI7L or ASFV-WT at an MOI of 0.1, and the viral titers and the ASFV genome copies were quantified at 48 hpi. The results showed that the ASFV-ΔI7L replication was significantly lower than that of ASFV-WT upon IFN-*γ* treatment (Fig 3C and 3D), indicating that pI7L counteracts the antiviral activity of IFN-*γ*. In addition, the transcriptional levels of ISGs in the ASFV-ΔI7L- or ASFV-WT-infected PAMs were examined using RT-qPCR. The results revealed that the transcriptional levels of *IRF1* (Fig 3E), *CXCL10* (Fig 3F), *SOCS1* (S4A Fig), *GBP1* (S4B Fig), and *CXCL9* (S4C Fig) in the ASFV-infected PAMs were consistent with the RNA-seq data at 20 hpi, further confirming that pI7L inhibits the IFN-*γ*-triggered JAK-STAT signaling pathway.

### pI7L inhibits the IFN-*γ*-triggered JAK-STAT signaling pathway

To further validate the involvement of pI7L in IFN-*γ*-triggered JAK-STAT signaling, a reporter assay of the *IRF1* promoter was performed in HEK293T cells. As expected, pI7L inhibited the activation of the *IRF1* promoter triggered by IFN-*γ* in a dose-dependent manner (Fig 3G). Furthermore, the transcriptional level of ISGs was examined following treatment with IFN-*γ* by RT-qPCR in the pI7L-expressing HEK293T cells. The results showed that the ectopically expressed pI7L remarkably inhibited the transcriptional levels of the *IRF1* (Fig 3H), *CXCL10* (Fig 3I), and *GBP1* (S4D Fig) induced by IFN-*γ*. Collectively, these findings clarify that pI7L negatively regulates the IFN-*γ*-triggered JAK-STAT signaling pathway.

### pI7L inhibits the JAK-STAT signaling pathway by targeting STAT1

**Fig 4:**
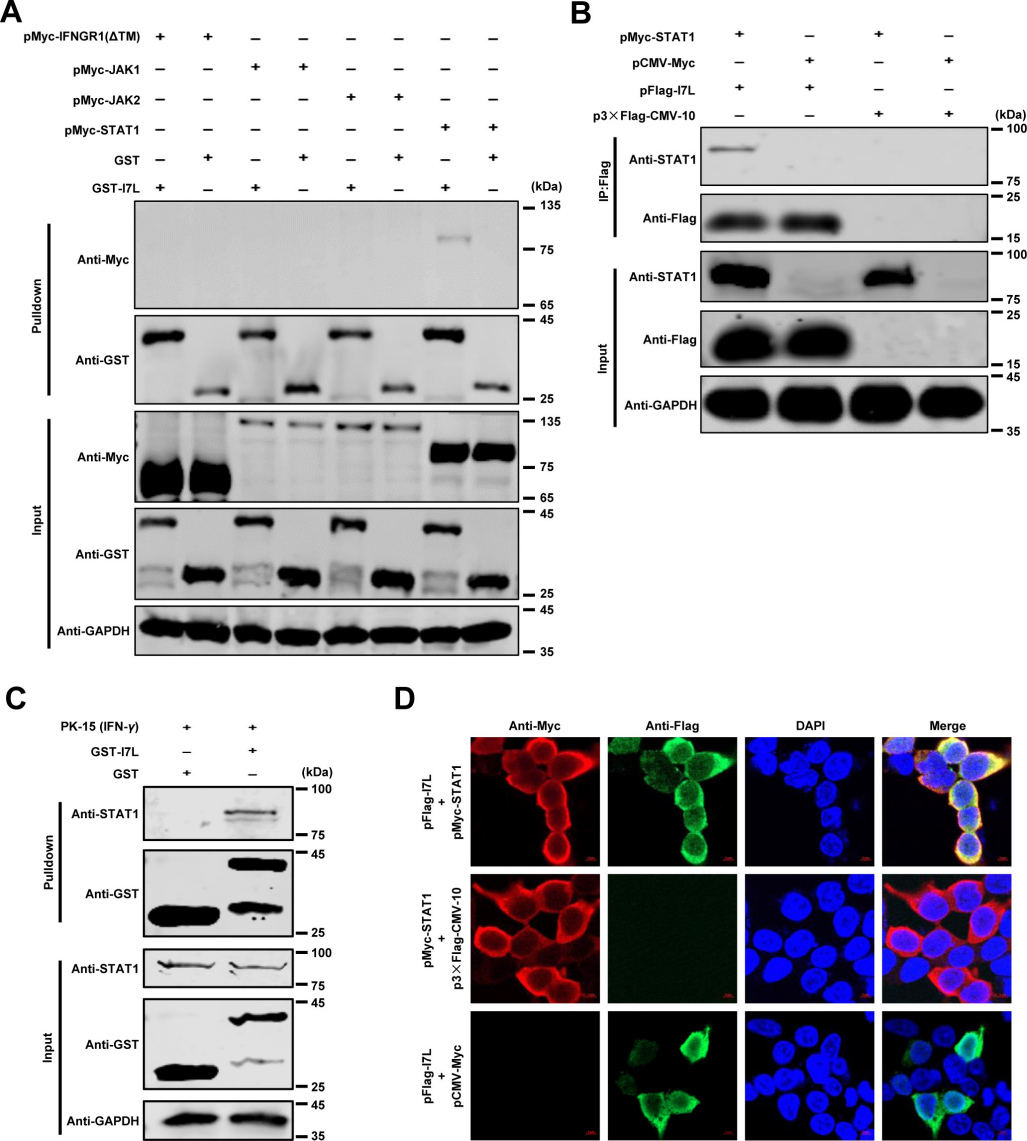
pI7L inhibits the IFN-*γ*-triggered JAK-STAT signaling pathway by targeting STAT1. (A–C) pI7L interacts with STAT1. HEK293T cells were seeded into 6-well plates and transfected with pMyc-IFNGR1(ΔTM), -JAK1, -JAK2, or -STAT1. At 48 hours posttransfection (hpt), the cells were collected and lysed with NP-40 buffer. The purified GST or GST-pI7L protein was used to pull down the crucial adaptors of the JAK-STAT pathway and analyzed by Western blotting using a mouse anti-GST or -Myc monoclonal antibody (MAb) (A). HEK293T cells were cotransfected with pFlag-I7L and pMyc-STAT1. At 48 hpt, the lysates were collected and incubated with anti-Flag beads, and then the bound proteins were examined by Western blotting using a rabbit anti-Flag, -Myc, or -GAPDH MAb (B). PK-15 cells were treated with IFN-*γ* (100 ng/ml) for 12 hours and lysed with NP-40 buffer. The purified GST or GST-pI7L protein was used to pull down STAT1 and analyzed by Western blotting using a mouse anti-GST or -STAT1 MAb (C). (D) pI7L is colocalized with STAT1. For the confocal microscopy, the HEK293T cells were cotransfected with pFlag-I7L and pMyc-STAT1. At 24 hpt, the cells were incubated with a mouse anti-Flag or rabbit anti-Myc MAb and Alexa Fluor 488 (green)- or 594 (red)-conjugated secondary antibodies, respectively. The cell nuclei (blue) were stained with 4’,6-diamidino-2-phenylindole (DAPI). Bars, 5 *μ*m.

To identify the adaptors involved in the function of pI7L in the JAK-STAT pathway, a glutathione S-transferase (GST) pulldown assay was performed. The GST-I7L or GST protein was incubated with the Myc-tagged adaptors including IFNGR1(ΔTM), JAK1, JAK2, or STAT1. The results indicated that pI7L specifically interacted with STAT1 but not with other adaptors (Fig 4A). In addition, a co-immunoprecipitation (co-IP) assay indicated that pI7L interacted with STAT1 (Fig 4B). For the endogenous pulldown assay, the GST-I7L or GST protein was incubated with the lysates of the PK-15 cells treated with IFN-*γ*, further confirming that pI7L was associated with STAT1 *in vitro* (Fig 4C). Consistently, the colocalization of pI7L with STAT1 was mainly observed in the cytoplasm (a colocalization coefficient of 0.90) by laser confocal microscopy (Fig 4D). Collectively, these results suggest that pI7L is associated with the JAK-STAT signaling at the STAT1 level.

### pI7L inhibits the phosphorylation and blocks the nuclear translocation of STAT1

**Fig 5:**
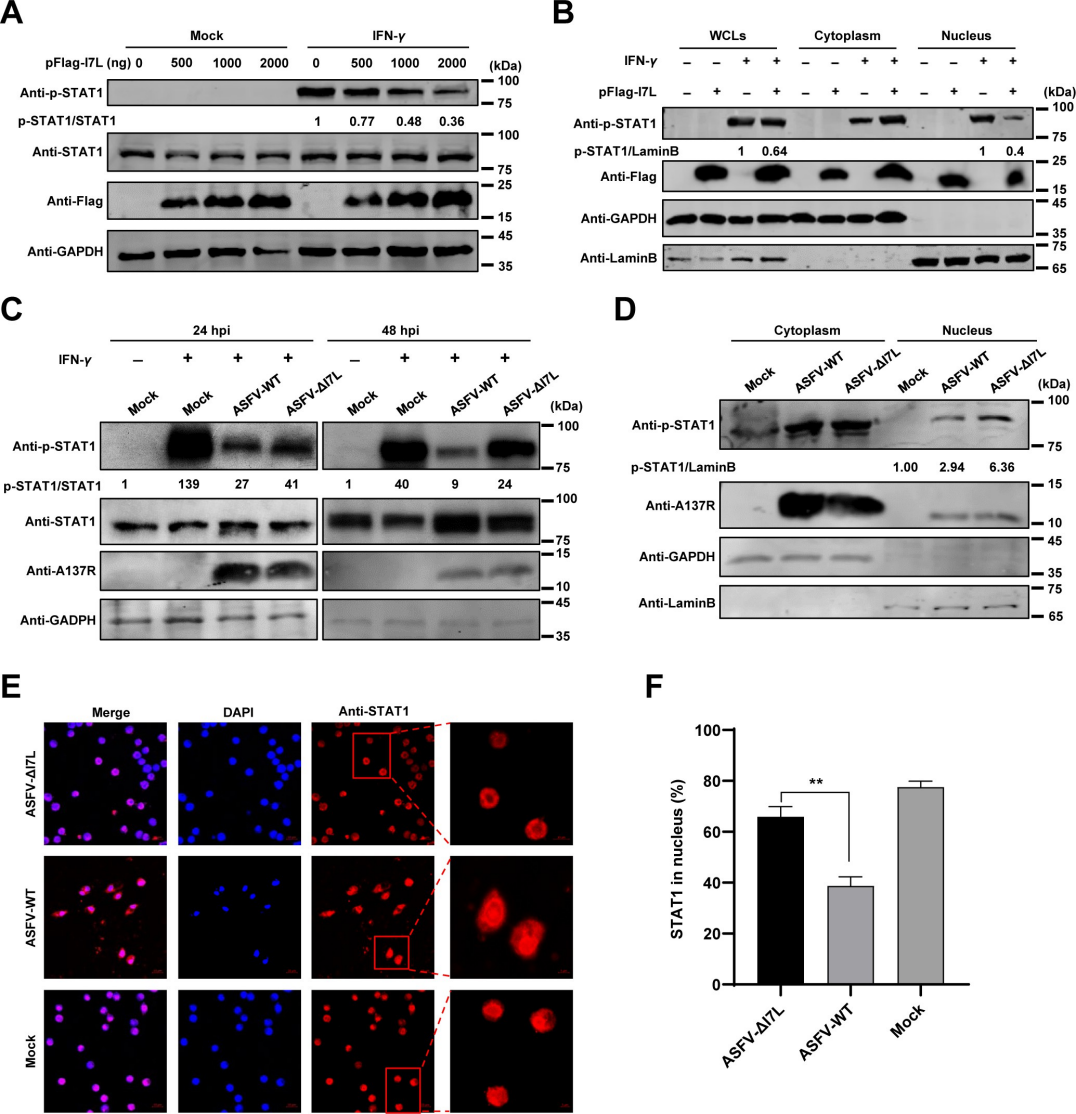
pI7L inhibits STAT1 phosphorylation and blocks its nuclear translocation. (A and B) pI7L inhibits the phosphorylation and the nuclear translocation of STAT1. HEK293T cells grown in 12-well plates were transfected with pFlag-I7L (500, 1000, or 2000 ng). At 24 hours posttransfection, the cells were treated with IFN-*γ* (100 ng/ml) or mock-treated for another 30 minutes, then the cells were lysed and the phosphorylation of STAT1 were examined by Western blotting using a rabbit anti-p-STAT1 (the phosphorylated STAT1), -STAT1, -Flag, or -GAPDH monoclonal antibody (MAb) (A). The pFlag-I7L–transfected cells were lysed and fractionated into cytoplasmic and nuclear fractions, and the p-STAT1 in the cytoplasmic and nuclear compartments were analyzed by Western blotting. Lamin B1 and GAPDH were used as nuclear and cytosolic markers, respectively. WCLs, whole cell lysates (B). (C–F) Deletion of the *I7L* gene promotes the phosphorylation and increases the nuclear translocation of STAT1. Primary porcine alveolar macrophages (PAMs) seeded into 12-well plates were infected with ASFV-ΔI7L or ASFV-WT at a multiplicity of infection (MOI) of 1 for 24 or 48 hours, followed by treatment with IFN-*γ* (100 ng/ml) for 30 minutes. The cells were lysed and the phosphorylation of STAT1 were analyzed by Western blotting using rabbit anti-p-STAT1, -STAT1, -A137R, or, -GAPDH MAbs (C). PAMs were mock-infected or infected with ASFV-ΔI7L or ASFV-WT at an MOI of 1 for 48 hours. The cells were lysed and fractionated into cytoplasmic and nuclear fractions, and the p-STAT1 in the cytoplasmic and nuclear compartments were analyzed by Western blotting. Lamin B1 and GAPDH were used as nuclear and cytosolic markers, respectively (D). PAMs were infected with ASFV-ΔI7L or ASFV-WT (MOI = 1) for 24 hours, and then treated with IFN-*γ* (100 ng/ml) for another 30 minutes. The subcellular localization of STAT1 (red) or cell nuclei (blue) was examined by laser confocal microscopy. Bars, 5 *μ*m (E). The cells with STAT1 nuclear translocation were counted from 100 cells per condition from different view fields (F). Error bars denote the standard errors of the means. All the data were analyzed using the Student’s *t* test. **, *P <* 0.01.

It has been shown that the phosphorylation at the Y701 residue leads to the homodimerization and nuclear translocation of STAT1, which play pivotal roles in the activation of the JAK-STAT signaling pathway [28]. Therefore, we examined the effects of pI7L on the phosphorylation and nuclear translocation of STAT1. The results showed that the STAT1 phosphorylation was inhibited by the ectopically expressed pI7L in a dose-dependent manner (Fig 5A), and the nuclear translocation of the phosphorylated STAT1 (p-STAT1) was significantly decreased in the pFlag-I7L-transfected cells (Fig 5B). In the context of ASFV infection, compared with ASFV-WT, ASFV-ΔI7L infection remarkably enhanced the STAT1 phosphorylation upon IFN-*γ* treatment at 24 and 48 hpi (Fig 5C), and the nuclear translocation of p-STAT1 was significantly increased in the ASFV-ΔI7L-infected PAMs (Fig 5D). Furthermore, the subcellular localization of STAT1 in the ASFV-ΔI7L-infected PAMs was determined by laser confocal microscopy (Fig 5E). The results showed that approximately 80% of STAT1 underwent nuclear translocation in the non-infected cells upon IFN-*γ* stimulation. Following ASFV-WT infection, only 40% of STAT1 was detected in the nucleus; however, more than 66% of STAT1 was detected in the nucleus upon ASFV-ΔI7L infection (Fig 5F). Collectively, these data suggest that the ASFV pI7L inhibits the phosphorylation and nuclear translocation of STAT1.

### The SH2 domain of STAT1 specifically recognizes pTyr of pI7L

**Fig 6:**
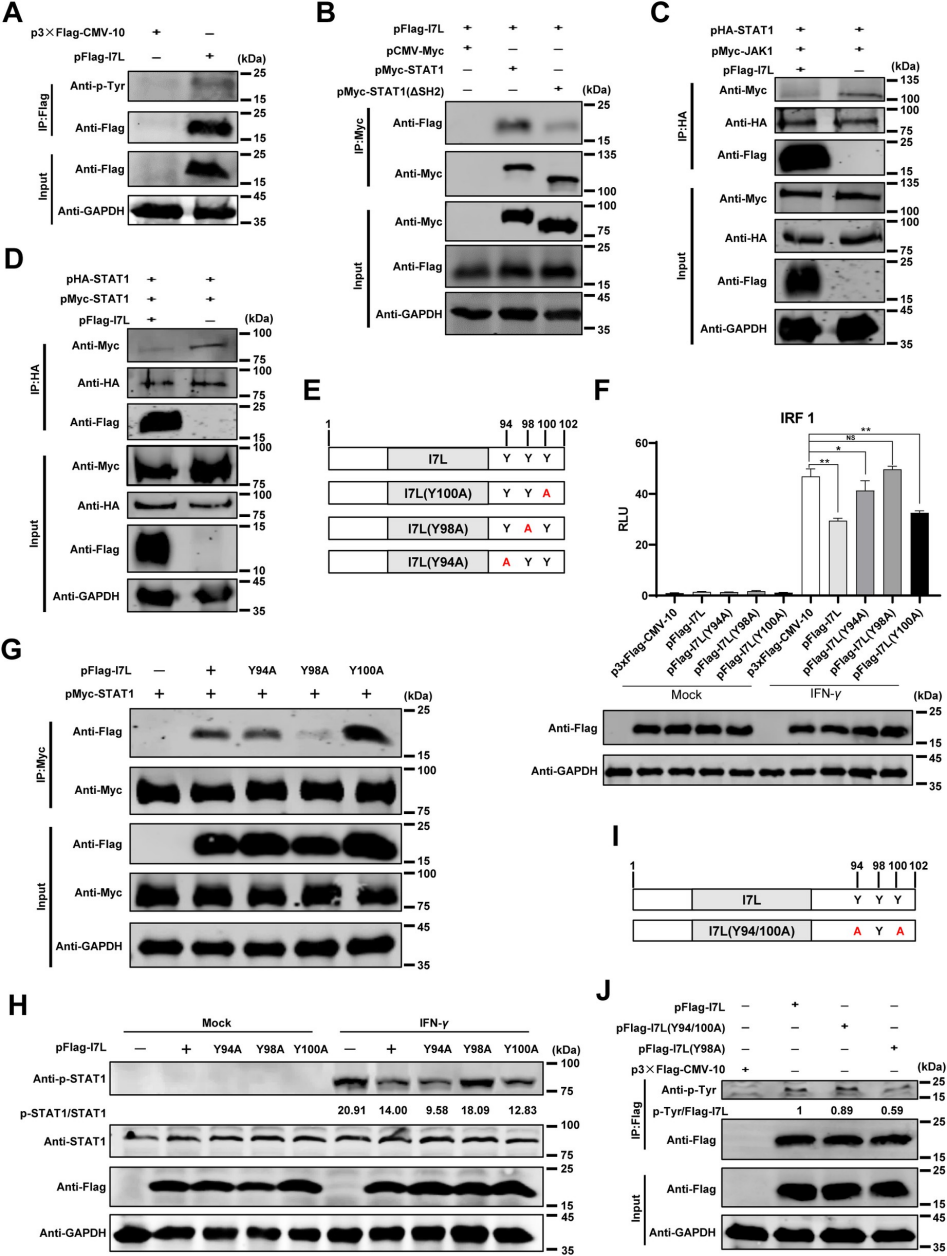
pI7L inhibits the phosphorylation of STAT1 depending on its tyrosine at position 98. (A) The phosphorylation modification occurs at the tyrosine residue of pI7L. HEK293T cells were transfected with pFlag-I7L or p3×Flag-CMV-10. At 24 hours posttransfection (hpt), the lysates were collected and incubated with anti-Flag beads, and then the bound proteins were examined by Western blotting using a rabbit anti-phosphotyrosine monoclonal antibody (MAb). (B) STAT1 interacts with pI7L depending on its SH2 domain. HEK293T cells were cotransfected pFlag-I7L with pMyc-STAT1 or pMyc-STAT1(ΔSH2). Co-IP assay was performed at 48 hpt using the anti-Flag beads. (C and D) pI7L inhibits the interaction between JAK1-STAT1 and STAT1-STAT1. HEK293T cells were cotransfected pFlag-I7L, pHA-STAT1 with pMyc-JAK1 (C) or pMcy-STAT1 (D). At 48 hpt, the lysates were collected and incubated with anti-HA beads, and then the bound proteins were checked by Western blotting using rabbit anti-Flag, -Myc, -HA, or -GAPDH MAb. (E) A schematic illustration of the tyrosine mutations in the C-terminus of pI7L. (F) pI7L(Y98A) fails to inhibit the activation of the *IRF1* promoter. HEK293T cells grown in 24-well plates were transfected with p3xFlag-CMV-10, pFlag-I7L, -I7L(Y94A), -I7L(Y98A), or -I7L(Y100A) (0.5 μg each) together with the promoter reporter plasmid pIRF1-Fluc (100 ng) and the internal reference reporter plasmid pSV40-Rluc (10 ng), respectively. At 24 hpt, the transfected cells were treated with IFN-*γ* (20 ng/ml) or mock-treated for another 12 hours, and then the cells were lysed and the reporter activity was analyzed with a dual-luciferase assay kit. (G) pI7L interacts with STAT1 depending on the tyrosine at position 98. HEK293T cells were cotransfected p3xFlag-CMV-10, pFlag-I7L, -I7L(Y94A), -I7L(Y98A), or -I7L(Y100A) with pMcy-STAT1. At 48 hpt, the co-IP assay was performed. (H) pI7L(Y98A) does not inhibit the phosphorylation of STAT1. HEK293T cells grown in 12-well plates were transfected with p3xFlag-CMV-10, pFlag-I7L, -I7L(Y94A), -I7L(Y98A), or -I7L(Y100A). At 24 hpt, the cells were treated with IFN-*γ* (100 ng/ml) or mock-treated for another 30 minutes, then the cells were lysed and the phosphorylation of STAT1 was checked by Western blotting using rabbit anti-p-STAT1 (the phosphorylated STAT1), -STAT1, -Flag, or -GAPDH MAb. (I) A schematic illustration of the tyrosine mutations of pI7L(Y94/100A). (J) The phosphorylation occurs at the tyrosine residue at position 98 of pI7L. HEK293T cells were transfected with pFlag-I7L, pFlag-I7L(Y94/100A), or p3×Flag-CMV-10. At 24 hpt, the lysates were collected and incubated with anti-Flag beads, and then the bounded proteins were checked by Western blotting using a rabbit anti-phosphotyrosine MAb. All the data were analyzed using the one-way ANOVA. Error bars denote the standard errors of the means. *, *P* < 0.05; **, *P* < 0.01; ns, not significant.

The SH2 domain of STAT1 specifically recognizes the pTyr-containing ligand proteins [33], and our findings have revealed the interaction between pI7L and STAT1. Subsequently, we investigated whether pI7L undergoes tyrosine phosphorylation and whether the pI7L-STAT1 interaction depends on the SH2 domain of STAT1. The co-IP results demonstrated that pI7L underwent tyrosine phosphorylation modification, as detected by a rabbit anti-phosphotyrosine monoclonal antibody (MAb) (Fig 6A), and STAT1 interacts with pI7L depending on its SH2 domain (Fig 6B).

### pI7L inhibits the phosphorylation and homodimerization of STAT1 depending on the Y98 residue

It has been demonstrated that JAK1 plays an important role in the IFN-*γ*-triggered JAK-STAT signaling pathway, where it interacts with STAT1 and directly phosphorylates STAT1 at the Y701 residue [28]. We examined whether pI7L affects the interaction of JAK1-STAT1 and STAT1-STAT1. The co-IP results revealed that the interaction of STAT1-JAK1 (Fig 6C) or STAT1-STAT1 (Fig 6D) was inhibited by the ectopically expressed pI7L, indicating that pI7L competes with JAK1 for binding to STAT1, thereby inhibiting the phosphorylation and homodimerization of STAT1.

The SH2 domain specifically recognizes pTyr-containing ligand proteins, which are highly dependent on the aa sequence directly C-terminal to the pTyr [24]. To investigate the crucial tyrosine residue in pI7L responsible for the pI7L-STAT1 interaction, we constructed a series of plasmids expressing the mutated pI7L (Fig 6E). To test which tyrosine residue of pI7L participates in the regulation of the IFN-*γ*-triggered JAK-STAT signaling pathway, the *IRF1* reporter assays were performed. The results showed that the pI7L mutant pI7L(Y98A) did not suppress the activation of the *IRF1* promoter induced by IFN-*γ* (Fig 6F). Additionally, we also determined that pI7L, pI7L(Y94A), and pI7L(Y100A) interacted with STAT1 (Fig 6G) and inhibited the phosphorylation of STAT1 (Fig 6H), but not pI7L(Y98A). Furthermore, we generated a pI7L(Y94/100A) mutant (with the Y residues at positions 94 and 100 mutated as A) (Fig 6I), and confirmed that the phosphorylation of pI7L(Y98A) was significantly lower than that of pI7L or pI7L(Y94/100A) by co-IP assay using an anti-phosphotyrosine MAb, suggesting that the Y98 residue of pI7L is responsible for its tyrosine phosphorylation (Fig 6J). Collectively, these findings suggest that pI7L inhibits the IFN-*γ*-triggered JAK-STAT signaling pathway by antagonizing the phosphorylation and homodimerization of STAT1, depending on the Y98 residue of pI7L.

### The *I7L* gene is associated with ASFV pathogenicity *in vivo*

**Fig 7:**
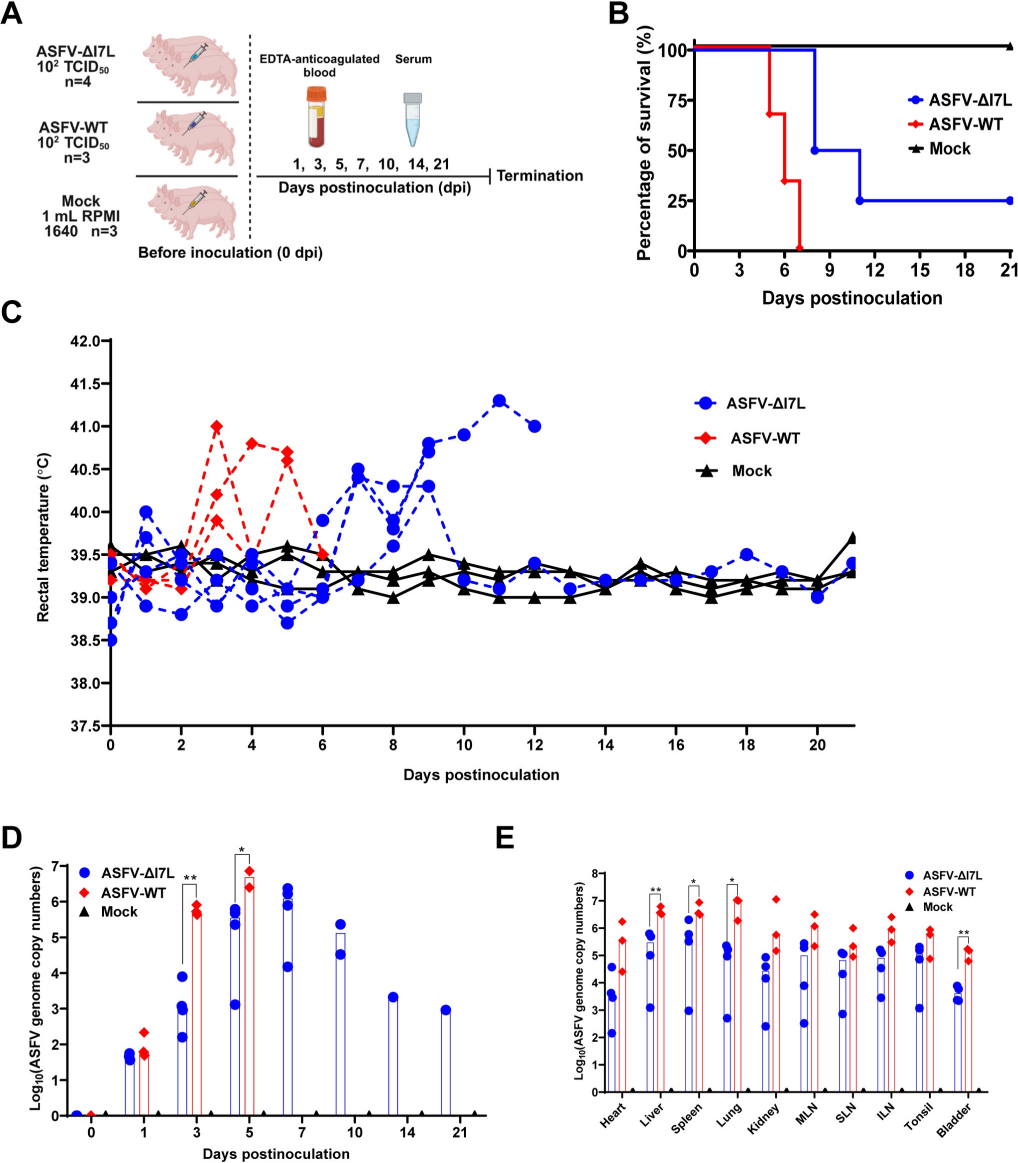
The *I7L* gene is involved in ASFV pathogenicity in pigs. (A) Schematic diagram of the pig inoculation experiment. The pigs were inoculated intramuscularly with ASFV-ΔI7L (*n* = 4, 10^2.0^ HAD_50_/pig) or ASFV-WT (*n* = 3, 10^2.0^ HAD_50_/pig) or mock inoculated with RPMI 1640 (*n* = 3, 1 mL/pig). The sera and the anticoagulated blood samples were collected at 0, 1, 3, 5, 7, 10, 14, and 21 days postinoculation. Created with BioRender.com. (B–E) pI7L is involved in ASFV pathogenicity in pigs. The survival rates (B) and rectal temperatures (C) of different groups were recorded. The viremia (D) and tissue viral loads (E) of each pig in different groups were quantified by a quantitative real-time PCR. MLN, mesenteric lymph node; SLN, submandibular lymph node; ILN, inguinal lymph node. All the data were analyzed using the Student’s *t* test. Error bars denote the standard errors of the means. *, *P* < 0.05; **, *P* < 0.01; ns, not significant.

SY18ΔI7L-I11L, an attenuated ASFV mutant with the *I7L-I11L* genes deleted, induces more IFN-*γ* than does the parental strain [16], and our findings clarify that pI7L negatively regulates the IFN-*γ*-triggered JAK-STAT signaling pathway via inhibiting the phosphorylation and homodimerization of STAT1 *in vitro*, suggesting that pI7L may contribute to the pathogenicity of ASFV. To evaluate the viral pathogenicity of ASFV-ΔI7L, we conducted a virulent challenge experiment in pigs. The pigs were inoculated intramuscularly (i.m.) with ASFV-ΔI7L or ASFV-WT at a dose of 10^2.0^ HAD_50_ or with RPMI 1640 (mock group) (Fig 7A). The disease progression was monitored for 21 d. All three pigs in the mock group presented no ASF-specific clinical signs and maintained normal body temperatures through the observation period. The pigs inoculated with ASFV-WT or ASFV-ΔI7L showed a mortality of 100% (3/3) or 75% (3/4), respectively (Fig 7B). After challenge with the virulent ASFV strain, all the three infected pigs developed fever at 3 days postinoculation (dpi), with body temperatures gradually increased to 41.8°C before death at 7 dpi. Conversely, three pigs in the ASFV-ΔI7L-inoculated group developed fever at 7 dpi and died at 8 and 11 dpi, one pig had a transient low fever before returning to normal temperature (Fig 7C). The ASFV-ΔI7L-inoculated pigs had lower ASFV genome copies in the anticoagulant blood samples compared with the ASFV-WT-inoculated pigs (Fig 7D). Additionally, the tissue samples from the liver, spleen, lung, and bladder of the ASFV-ΔI7L-inoculated pigs showed lower viral loads compared with those of the ASFV-WT-infected pigs (Fig 7E). Taken together, ASFV-ΔI7L exhibits reduced virulence compared with ASFV-WT in pigs, indicating that pI7L is involved in ASFV pathogenicity *in vivo*.

### ASFV-ΔI7L induces higher immune responses than does ASFV-WT in pigs

**Fig 8:**
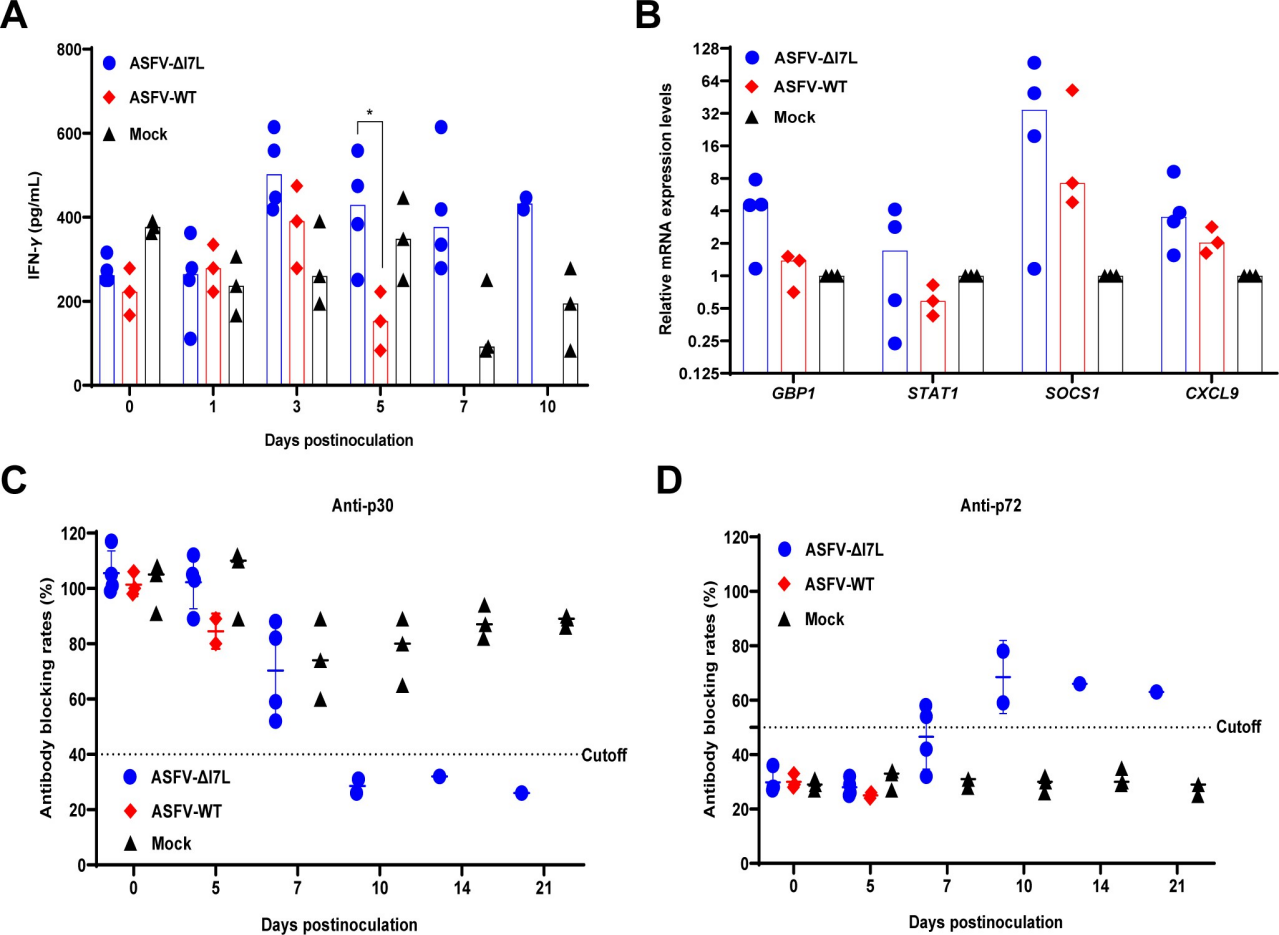
ASFV-ΔI7L induces higher immune responses than does ASFV-WT in pigs. (A and B) The ASFV-ΔI7L infection enhances the production of IFN-*γ* and IFN-*γ*-stimulated genes in pigs. The translational level of IFN-*γ* in the serum samples (A) and the mRNA transcriptional levels of *GBP1*, *STAT1*, SOCS1, and *CXCL9* in the lung (B) were measured by ELISA kits and a reverse transcription-quantitative PCR, respectively. (C and D) The ASFV-ΔI7L infection induces serum antibodies in the inoculated pigs. The anti-p72 (C) or -p30 (D) antibodies in the ASFV-ΔI7L- or ASFV-WT-inoculated pigs were tested by ELISA kits. Error bars denote the standard errors of the means. All the data were analyzed using the Student’s *t* test. *, *P* < 0.05; ns, not significant.

The *I7L*-*I11L* genes-deleted ASFV induced a higher level of IFN-*γ* production in pigs than did the parental ASFV strain [16]. Hence, we attempted to explore whether the ASFV-ΔI7L infection enhances IFN-*γ* production *in vivo*. We collected serum samples at 0, 1, 3, 5, 7, and 10 dpi to evaluate the levels of IFN-*γ*. The pigs infected with ASFV-ΔI7L produced elevated IFN-*γ* levels compared to those infected with ASFV-WT at 3, 5, and 7 dpi (Fig 8A). Furthermore, the transcription levels of the ISGs, including *GBP1*, *STAT1*, *SOCS1*, and *CXCL9* in the lungs derived from ASFV-ΔI7L-infected pigs were higher than those of their parental ASFV-WT-infected counterparts (Fig 8B).

Upon ASFV infection, anti-p30 and -p72 antibodies are important parameters for evaluating humoral immune responses and protection. To assess the immune response of pigs to ASFV-ΔI7L, we collected serum samples at 0, 5, 7, 10, 14, and 21 dpi to evaluate the circulating antibodies using ELISA. Anti-p30 (a blocking rate below 40% is considered positive) (Fig 8C) and anti-p72 (a blocking rate above 50% is considered positive) (Fig 8D) antibodies were detected in the ASFV-ΔI7L-infected pigs at 10 and 7 dpi, respectively, indicating that the partially attenuated ASFV-ΔI7L induced better antibody responses. In contrast, no anti-p72 and anti-p30 antibodies were detected in the indicated sera obtained from the mock- or ASFV-WT-inoculated pigs. Overall, these findings suggest that the pathogenicity of ASFV-ΔI7L in pigs is partially attenuated compared with ASFV-WT, as evidenced by increasing levels of circulating antibodies and IFN-*γ* production.

## Discussion

**Fig 9:**
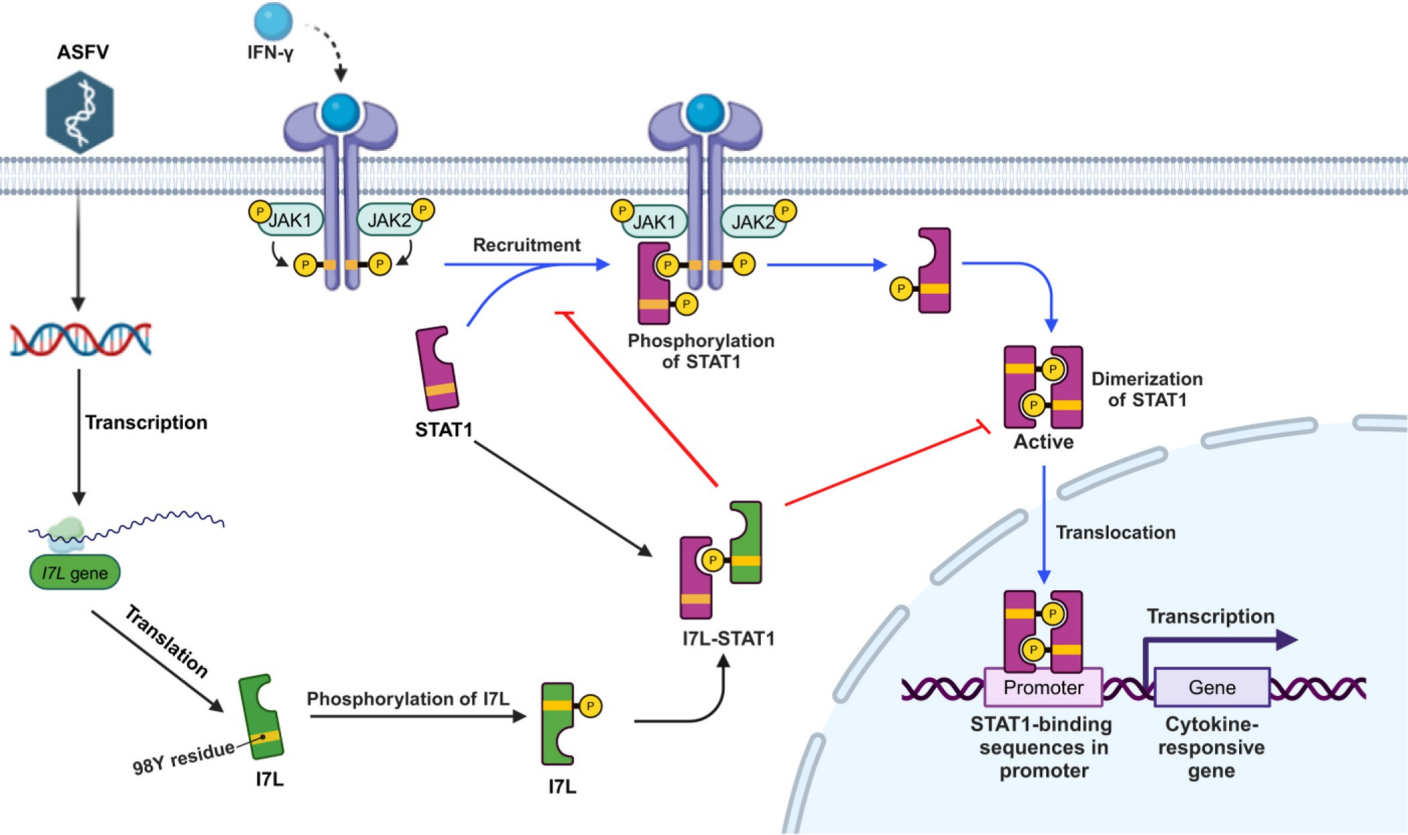
A schematic model of the ASFV pI7L negatively regulating the JAK-STAT signaling pathway targeting STAT1 upon ASFV infection. The ASFV pI7L can inhibit the IFN-*γ*-triggered JAK-STAT signaling pathway by targeting STAT1. pI7L interacts with STAT1 and inhibits the phosphorylation and homodimerization of STAT1 depending on the tyrosine at position 98 as well as the nuclear translocation of STAT1. Created with BioRender.com.

In this study, we demonstrated that pI7L is not associated with the replication of ASFV in PAMs (Fig 1), whereas, it is involved in ASFV pathogenicity *in vivo* (Figs 7 and 8). Interestingly, our findings indicated that the DEGs in the PAMs infected with ASFV-ΔI7L were mainly involved in antiviral immune responses induced by IFN-*γ* (Fig 2), and we further confirmed that the pI7L suppresses the IFN-*γ*-triggered JAK-STAT signaling pathway (Fig 3). Notably, we elucidated the immunoevasion mechanism by which pI7L interacts with STAT1 and inhibits the IFN-*γ*-triggered JAK-STAT signaling pathway through antagonizing the phosphorylation and homodimerization of STAT1 depending on the Y98 residue of pI7L and the SH2 domain of STAT1 (Figs 4–6). Taken together, these findings suggest that pI7L is an immunoevasion factor of ASFV, regulating the IFN-*γ*-triggered JAK-STAT signaling pathway (Fig 9).

Since the reemergence of ASF in Europe in 2007, the disease has spread widely across major pig-producing countries in Europe and Asia, causing significant economic losses to the global pig industry [34]. Theoretically, safe and effective vaccines are among of the most effective strategies to prevent and control ASF outbreaks. However, multiple factors continue to hinder the development and application of ASF vaccines [35,36]. One significant limitation is the lack of production cell lines for virus passage, which restricts the development of ASF vaccines and the functional study of the ASFV-encoded proteins. ASFV is primarily propagated in primary porcine monocytes and macrophages. Remarkably, our previous study showed that ASFV could replicate at a low level in HEK293T cells, leading to the development of an HEK293T cells-adapted virus strain through continuous passage [12]. Similar to other cells-adapted ASFV stains like ASFV-G/V60/80/110 and BA71V [13], the *I7L* gene is lost in the right variable regions of the ASFV-P61 genome. Therefore, we speculate that the deletion of the *I7L* gene is critical to the adaptation of ASFV in HEK293T cells. It has been reported that the adaptation of ASFV to cell lines is often accompanied by the deletion of the left multiple genes family (MGF), such as the deletion of *MGF360* and *MGF505* in the left variable region of the genome, which may lead to the adaptation of the BA71 strain to Vero cells [11]; Similarly, the genes of the MGF360 family were predominantly absent within the left variable region of the ASFV-G/V80 genome [13]. Thus, the determinants underlying cell adaptation of ASFV are not single but multiple genes. Considering that the adaptation of ASFV to cell lines may be determined by multiple genes, future research should focus on identifying key genes and elucidating the molecular mechanisms underlying cellular adaptability.

A recent study showed that the deletion of large fragments of the *I7L-I11L* genes in the right variable region of the ASFV-SY18 genome, including the *I7L*, *I8L*, *I9R*, *I10L*, and *I11L* genes, did not affect the replication of ASFV in PAMs [16]. In this study, we demonstrated that the growth kinetics of ASFV-ΔI7L were similar to those of ASFV-WT in PAMs (Fig 1D and 1E), indicating that the deletion of the *I7L* gene does not impair the replication of ASFV in PAMs, consisting with the finding for the *I7L-I11L* genes deletion. The animal experiment demonstrated that the SY18ΔI7L-I11L mutant induced increased IFN-*γ* production in pigs. Our findings revealed that both the transcription and translation of IFN-*γ* were significantly enhanced upon ASFV-ΔI7L infection *in vitro* and *in vivo*, indicating that pI7L might exert pivotal roles in the regulation of IFN-*γ* production, which warrants further research. Furthermore, we confirmed that pI7L inhibits the IFN-*γ*-triggered JAK-STAT signaling pathway, albeit with a modest effect on this response. In fact, ASFV encodes many similar functional proteins antagonizing the IFN-*γ*-mediated JAK-STAT1 axis, such as the MGF505-7R protein. Due to the existence of functional compensation, pI7L inhibition of IFN-*γ* responses may have a modest effect on ASFV infection. Although our results demonstrated that pI7L negatively regulate the JAK-STAT signaling pathway by targeting STAT1, we cannot exclude the possibility that the deletion of the *I7L* gene may impact the expression of other ASFV proteins which also inhibit the JAK-STAT signaling pathway.

The SY18ΔI7L-I11L mutant leads to attenuated virulence [16], suggesting that one or more of the I7L, I8L, I9R, I10L, and I11L proteins may be involved in the immune escape of ASFV, but the underlying immunoevasion mechanism remains largely unknown. Previously, we demonstrated that pI10L inhibits the NF-*κ*B signaling pathway by targeting IKK*β* [37]. In this study, we clarified that pI7L negatively regulates the IFN-*γ*-triggered JAK-STAT signaling pathway via inhibiting the phosphorylation and homodimerization of STAT1 depending on its Y98 residue. So far, the functions of the I8L, I9R, and I11L proteins remain unclear, and we will further elucidate the biological functions of these proteins in the future.

Deletion of the *I7L-I11L* genes results in the attenuation of the ASFV SY18 strain [16], but which genes are virulence-related genes remains elusive. Deletion of the *I8L* gene in the ASFV-G strain (genotype II) genome does not affect viral replication either *in vitro* or *in vivo*, indicating that the *I8L* gene is not a virulence-related gene of ASFV [38]. The I11L protein is a late-transcribed protein, and deletion of the *I11L* gene from the Malawi Lil-20/1 strain (genotype VIII) genome did not affect the replication and virulence of ASFV [39]. However, deletion of the *I11L* gene from the ASFV SY18 strain (genotype II) genome does not inhibit the viral replication *in vitro* but significantly reduces the pathogenicity *in vivo* [40], indicating that the *I11L* gene is a conditionally virulence-related gene, depending on the genetic background of the strains. The pI10L of ASFV shares homology with the p22 protein encoded by the *KP177R* gene. Notably, deletion of the *KP177R* gene from the ASFV-G strain genome does not affect the replication and the pathogenicity of ASFV [41]. Similarly, deletion of the *I10L* gene from the ASFV SY18 strain genome does not affect the replication or the pathogenicity of ASFV [40]. Furthermore, deletion of the *I10L* gene from the ASFV SY18 strain genome did not affect the replication and the virulence of ASFV [40]. This study confirmed that pI7L is involved in the virulence of ASFV. Even though the mortality of the ASFV-ΔI7L infection group is 75%, the body temperature and viral replication were significantly reduced and the death time was delayed compared with those of ASFV-WT in pigs, indicating that pI7L is associated with ASFV pathogenicity *in vivo*. Similarly, the *I7L* gene from the ASFV SY18 strain genome is associated with ASFV virulence [40]. But we found that ASFV-ΔI7L remained partially pathogenic to pigs, indicating that the *I7L* gene contributes to virus virulence in combination with other gene(s).

Recently, integrative multi-omics analyses have been widely used to study gene function and reveal changes in host metabolic pathways, among which transcriptome sequencing is the most widely used analysis technique. Since the *I7L* gene of ASFV remains functionally uncharacterized, this study aims to explore the biological functions of pI7L in the ASFV replication cycle through transcriptome sequencing. The DEGs in the PAMs infected with ASFV-ΔI7L were identified to be associated with IFN-*γ* and downstream ISGs using RNA-seq analysis, indicating that pI7L may be associated with the IFN-*γ*-triggered JAK-STAT signaling pathway. Furthermore, we found that the DEGs were involved in the host immune response, inflammatory response, regulation of the phosphatidylinositol 3-kin (PI-3K) signaling pathway, RIG-I-like receptor signaling pathway, suggesting that pI7L is a multifunctional protein. We will further analyze its other biological functions in the viral replication cycle in the future.

The SH2 domain, consisting of about 100 aa residues, is widely found in tyrosine kinases, phosphatases, signaling adaptor proteins and transcription factors, and these proteins participate in the regulation of the corresponding signal transduction pathways, such as IFN, PI3K, and JAK-STAT signaling pathways [19,20]. These pathways play crucial roles in a variety of biological processes, including antiviral immune responses, differentiation, hematopoiesis, and cell growth [20]. The human genome encodes approximately 111 tyrosine kinases and 121 SH2 domains [42,43]. Besides eukaryotes, SH2 proteins have been identified in some bacteria [44], and viruses [45,46]. Many viruses encode SH2-containing proteins, such as the tyrosine kinase v-Src, the adaptor protein v-Crk, and the ubiquitin ligase v-Cbl [44,47], and these SH2-containing viral proteins play key roles in hijacking the phosphotyrosine signaling pathways to escape the host immune responses [47]. In this study, we focused on the ASFV *I7L* gene encoding the 102-aa pI7L, which is similar to the aa number of the SH2 domain. Using the online software I-TASSER (https://zhanggroup.org/I-TASSER/output/S757713/) [48], we predicted that the ASFV pI7L contains an SH2 domain, which is close to the SH2 domain of target proteins in the PDB, such as the Syp tyrosine phosphatase (https://www.rcsb.org/structure/1AYD), phosphoinositide 3-kinase (https://www.rcsb.org/structure/1BFJ), cytoplasmic protein NCK2 (https://www.rcsb.org/structure/2CIA), growth factor receptor-bound protein-2 (https://www.rcsb.org/structure/1FHS), and tyrosine-protein kinase transforming protein (https://www.rcsb.org/structure/1BKL). Additionally, the aa 9–91 of pI7L was predicted to be an SH2 domain in Uniprot (https://www.ebi.ac.uk/interpro/protein/UniProt/A0A0C5B0D0/). Our data demonstrated that pI7L negatively regulates the IFN-*γ*-triggered JAK-STAT signaling pathway to escape the host immune response. Resolving the crystal structure of pI7L is essential to provide direct evidence for the involvement of the domain in immunoevasion.

In the IFN-*γ*-triggered JAK-STAT signaling pathway, activation of JAK1 leads to the phosphorylation of STAT1 at the Y701 residue [28], which forms STAT1 homodimers that are translocated to the nucleus and induce the transcription of various ISGs. Our study revealed that pI7L competes with JAK1 to bind STAT1 and, thereby inhibiting its phosphorylation and subsequent homodimerization. The SH2 domain specifically recognizes pTyr-containing ligand proteins, which is highly dependent on the aa sequence directly C-terminal to the pTyr residue. Our results showed that pI7L inhibits the IFN-*γ*-triggered JAK-STAT signaling pathway by antagonizing the phosphorylation and homodimerization of STAT1 depending on the Y98 residue of pI7L. However, how the tyrosine-protein kinase mediates the pI7L phosphorylation at the position 98 of tyrosine residue remains to be explored.

In the JAK-STAT signaling pathway, the SH2 domain of one STAT1 molecule specifically recognizing the phosphorylated tyrosine at position 701 (pY701) residue of another STAT1, and vice versa, the binding of two SH2-pY701 copies results in the formation of STAT1-STAT1 homodimers. In this study, our results indicated that the wild-type pI7L underwent tyrosine phosphorylation (Fig 6A) and the STAT1 interacts with pI7L depending on its SH2 domain (Fig 6B), indicating that the SH2 domain of STAT1 specifically recognizes the phosphorylated tyrosine of pI7L. Furthermore, we further demonstrated that the pI7L(Y94/100A) mutant underwent tyrosine phosphorylation (Fig 6J), and the Y98A mutation in pI7L significantly weakened but did not completely abolish its interaction with STAT1 (Fig 6G), indicating that the interaction of pI7L and STAT1 depends on the SH2 domain of STAT1 specifically recognizing the pTyr at position 98 of pI7L. Interestingly, deletion of the SH2 domain of STAT1 (Fig 6B) or the tyrosine residue at position 98 mutated as alanine of pI7L (Fig 6G) significantly weakened but did not completely abolish its interaction between STAT1 and pI7L, implying potential alternative interaction modes beyond dependence on the SH2 domain of STAT1 and the Y98 residue of pI7L. The protein structure prediction revealed that pI7L contains an SH2 domain which likely recognizes the phosphorylated tyrosine at position 701 of STAT1. However, the crystal structure of pI7L needs be resolved to provide direct evidence for interaction between STAT1 and pI7L.

To detect the expression of pI7L following ASFV infection, we tried to prepare mouse and rabbit polyclonal antibodies (PAbs) against pI7L to verify the functions of pI7L, but unfortunately, both PAbs failed to detect the expression of pI7L upon ASFV infection, suggesting that pI7L may possess low immunogenicity and that it is necessary to optimize the immunization dose, route, and adjuvant.

## Materials and methods

### Ethics statement

The animal experiment was conducted in compliance with the Animal Welfare Act and Guide for the Care and Use of Laboratory Animals, approved by the Laboratory Animal Welfare Committee of Harbin Veterinary Research Institute (HVRI) of the Chinese Academy of Agricultural Sciences (approval number 230724-01-GR).

### Biosafety statement and facilities

All the experiments with live ASFV were conducted within the animal biosafety level 3 (ABSL-3) facilities at HVRI.

### Virus strain, cells, and antibodies

The ASFV HLJ/2018 strain (GenBank accession no. MK333180.1) was propagated in PAMs [49]. PAMs were cultured with RPMI 1640 medium (catalog no. C11875500BT; Gibco) supplemented with 10% fetal bovine sera (FBS) (catalog no. 10099141; Gibco) and 2% antibiotics-antimycotics (catalog no. 15140122; Gibco) [12]. HEK293T or PK-15 cells were cultured in Dulbecco’s modified Eagle’s medium-high glucose (catalog no. C11995500BT; Gibco), supplemented with 10% FBS [50].

The rabbit anti-STAT1 (catalog no. 14994S) and -phospho-STAT1 (catalog no. 9167S) MAbs were purchased from Cell Signaling Technology. The mouse anti-Flag (catalog no. F1804), -Myc (catalog no. M4439), -glyceraldehyde-3-phosphate dehydrogenase (GAPDH) (catalog no. SAB1405848), -GST (catalog no. SAB4701016) MAbs, and rabbit anti-HA (catalog no. SAB4300603) PAb were purchased from Sigma-Aldrich. The rabbit anti-phosphotyrosine MAb (catalog no. PTM 702RM) was purchased from PTM Biolabs. The rabbit anti-lamin B1 (catalog no. 12987-1-AP) PAb was purchased from Proteintech. Alexa Fluor 488-conjugated goat anti-mouse IgG (H+L) (catalog no. 1674651) and Alexa Fluor 647-conjugated goat anti-rabbit IgG (H+L) (catalog no. 1692912) antibodies were purchased from Life Technology.

### Construction of plasmids

The ASFV *I7L* gene was cloned into the p3xFlag-CMV-10 vector (Sigma-Aldrich) to generate pFlag-*I7L*. The porcine *IFNGR1(ICD)*, *IFNGR1(ΔTM)*, *JAK1*, *JAK2*, and *STAT1* genes were amplified and cloned into the pCMV-Myc vector (Clontech) to generate pMyc-IFNGR1(ICD), pMyc-IFNGR1(ΔTM), pMyc-JAK1, pMyc-JAK2, and pMyc-STAT1, respectively. The primers for amplification of these genes are listed in S1 Table.

### Generation and identification of the *I7L*-deleted ASFV mutant

A recombinant transfer vector pOK12-p72EGFP-ΔI7L was constructed as described previously [32]. Briefly, the left and right arms were amplified by PCR and assembled to contain the EGFP marker by overlapping PCR. The expression cassette was then cloned into the linearized pOK12 vector to generate the recombinant transfer vector pOK12-p72EGFP-ΔI7L using the ClonExpress II One Step Cloning kit (catalog no. C112; Vazyme).

The *I7L*-deleted mutant ASFV-ΔI7L was generated by homologous recombination between the parental virus ASFV-WT genome and the recombination transfer vector by transfection and infection procedures in PAMs [51]. Briefly, PAMs were seeded into 6-well plates and transfected with 2 *μ*g of the transfer vector pOK12-p72EGFP-ΔI7L using X-tremeGENE HP. At 24 hours posttransfection (hpt), the cells were infected with ASFV-WT (MOI = 1) for 48 hours. The recombinant virus was purified by successively limiting dilution, and the purified ASFV-ΔI7L was identified by PCR using specific primers targeting those genes (S1 Table) and sequencing analysis.

### NGS analysis of the ASFV-ΔI7L genome

To rule out mutations in other genes during the purification process, the purified ASFV-ΔI7L was identified by NGS. Briefly, the ASFV-ΔI7L genome DNA was extracted as described above [32]. Full-length sequencing of the whole genome was performed using the Illumina HiSeq 2500 platform of Novogene (Beijing, China).

### Hemadsorption (HAD) assay

The HAD assay was used to evaluate the median tissue culture infectivity of ASFV [52]. Briefly, PAMs (5 × 10^4^) were seeded into 96-well plates, and infected with ASFV-ΔI7L or ASFV-WT at an MOI of 0.01. At 48 hpi, porcine red blood cells (5 × 10^5^) were added to each well, and the "rosettes" of red blood cells was observed using an optical microscope.

### Determination of ASFV-ΔI7L replication kinetics

To evaluate the replication kinetics of ASFV-ΔI7L, the growth curves of ASFV-ΔI7L and ASFV-WT were examined in PAMs. Briefly, PAMs seeded into 24-well plates were infected with ASFV-ΔI7L or ASFV-WT at an MOI of 0.01, and rinsed twice with phosphate buffer saline (PBS) and replaced with fresh medium at 2 hpi. Subsequently, the cell supernatants were harvested at 2, 12, 24, 48, 72, 96, and 120 hpi for virus titration in PAMs.

### TEM assay

The assay was conducted as described previously [32] with a slight modification. Briefly, PAMs were seeded into 6-well plates and infected with ASFV-WT or ASFV-ΔI7L at an MOI of 1, and the cells were harvested and fixed with 2% glutaraldehyde in PBS for 1 hour at 24 hpi. The samples were dehydrated, embedded, and stained according to standard procedures. The samples were analyzed on an H-7650 (Hitachi, Tokyo, Japan) operated at 80 kV.

### RNA-seq analysis

PAMs were infected with ASFV-ΔI7L or ASFV-WT at an MOI of 1. At 4, 12, and 20 hpi, the cells were harvested and subjected to RNA extraction using a TRIzol kit (Invitrogen, USA) according to the manufacturer’s instructions. Total RNA was qualified and quantified using the Nanodrop2000 device (Thermo Fisher Scientific, MA, USA), and integrity and concentration were measured using the RNA Nano 6000 assay kit of the Bioanalyzer 2100 system (Agilent Technologies, CA, USA). The mRNA was purified using the Dynabeads mRNA DIRECT kit (Invitrogen, MA, US) and 200 ng of the mRNA was converted into double-stranded cDNA (ds-cDNA). The ds-cDNA were subjected to heat-denatured and circularized by the splint oligo sequence to generate the single-stranded circle DNA, followed by rolling circle replication to create DNA nanoball (DNB) using the MGIeasy RNA Library Prep (MGI Tech, Shenzhen, China). The samples were sequenced using the MGI DNBSEQ-G400RS (MGI Tech, Shenzhen, China) at two 150-base paired-end reads.

### Analysis of sequencing data

Clean reads were acquired from raw data using the FASTQ_Quality_Filter tool of the FASTX-toolkit, and then mapped to the reference *Sus scrofa* genome (GCF_000003025.6_Sscrofa11.1). The unique mapped reads were used for further analysis. The DEGs were analyzed using the deseq2 [53], *P* value of 0.05 was set as thresholds for the DEGs. The GO and KEGG pathway enrichment associated with the DEGs was performed using the R package (ClusterProfiler) [54] and KOBAS 3.0 [55].

### Dual-luciferase reporter (DLR) assay

To analyze the IFN-*γ*-triggered JAK-STAT pathway by DLR assay, HEK293T cells grown in 24-well plates were transfected with pFlag-I7L or p3xFlag-CMV-10 (200 ng each) together with the promoter reporter plasmid pIRF1-Fluc (100 ng) and the internal reference reporter plasmid pSV40-Rluc (10 ng). At 24 hpt, the transfected cells were treated with IFN-*γ* (catalog no. RP0126S-025; Kingfisher) (20 ng/ml) or mock-treated for another 12 hours, and then the cells were lysed and the reporter activity was analyzed with a dual-luciferase assay kit (catalog no. E1910; Promega). The data were represented as relative expression levels of Fluc/Rluc.

### Real-time PCR

The ASFV genomic DNA in the cells or cell supernatants was extracted using the MagaBio Plus virus DNA purification kit (BioFlux) according to the manufacturer’s protocols. The ASFV genome copies were quantified by a quantitative real-time PCR (qPCR) on the QuantStudio system (Applied Biosystems, USA) as described previously [56].

The transcriptional levels of *IRF1*, *CXCL10*, *GBP1*, *CXCL9*, or *SOCS1* in HEK293T cells or PAM were quantified using RT-qPCR by the 2^−ΔΔCT^ method [57]. The transcriptional level of GAPDH was set as an internal loading control. The primers used for RT-qPCR are listed in S1 Table.

### Co-IP assay

HEK293T cells were cotransfected with pMyc-STAT1 and pFlag-I7L for the co-IP assay. At 48 hpt, the samples were lysed with the NP-40 lysis buffer containing protease inhibitor 1 mM PMSF (catalog no. ST506; Beyotime) for 30 minutes, and then centrifuged for 20 minutes at 4°C. The supernatants were denatured in 1 × sample loading buffer for 7 minutes at 100°C. For the co-IP assay, the supernatants were mixed with the respective primary antibodies in the presence of protein G-agarose (catalog no. 11243233001; Roche) at 4°C overnight, followed by Western blotting analysis [50].

### GST-pulldown assay

For the construction of the pGST-I7L plasmid, the *I7L* gene was cloned into the pGEX-6p-1 expression vector. The glutathione S-transferase (GST) or GST-I7L fusion protein was expressed in *Escherichia coli* BL21(DE3) cells, induced by the addition of isopropylthiogalactoside, and purified using the glutathione-sepharose 4B resin (catalog no. 10049253; GE Biosciences) according to the manufacturer’s instructions. Briefly, the soluble GST or GST-I7L was incubated with the resin for 5 hours at 4°C after centrifugation at 12,000 × *g* for 20 min. Subsequently, the purified GST or GST-pI7L fusion protein was mixed with lysates from cells transfected with pMyc-IFNGR1(ICD), pMyc-IFNGR1(ΔTM), pMyc-JAK1, pMyc-JAK2, or pMyc-STAT1 at 4°C overnight. After five times washes with PBS, the proteins pulled down by GST resin were analyzed by Western blotting using a rabbit anti-Myc or mouse anti-GST MAb.

### Confocal imaging

HEK293T cells seeded into glass-bottomed dishes were cotransfected with pMyc-STAT1 and pFlag-I7L (1 μg each) for 36 hours. At 36 hpt, the cells were stained with the indicated antibodies and the images were acquired using a Leica SP2 confocal system (Leica Microsystems) [50].

### Subcellular localization of endogenous STAT1

PAMs were infected with ASFV-WT or ASFV-ΔI7L at an MOI of 1 for 48 hours. Subsequently, the cells were stimulated with 20 ng/mL IFN-*γ* for another 12 hours. The subcellular localization of STAT1 was examined by confocal microscopy using a rabbit anti-STAT1 MAb (1:100). Furthermore, the nuclear translocation of STAT1 was checked using a nuclear and cytoplasmic protein extraction kit (catalog no. P0028; Beyotime).

### Pig inoculation experiment

Ten specific-pathogen-free pigs, aged 7 weeks and weighing between 11.5 and 13.5 kg, were obtained from the Laboratory Animal Center of HVRI. The pigs were randomly divided into three groups and inoculated i.m. with the indicated ASFV strain to investigate the pathogenesis of ASFV-ΔI7L, with four pigs inoculated with ASFV-ΔI7L (10^2.0^ HAD_50_/pig); three pigs inoculated with ASFV-WT (10^2.0^ HAD_50_/ pig); three pigs inoculated with RPMI 1640 (1 mL/pig). Daily monitoring was performed for each pig, including the rectal temperatures and the clinical signs (lethargy, anorexia, depression, vomiting, fever, skin hemorrhages, bloody diarrhea, and joint swelling). EDTA-anticoagulated blood or sera were collected from all the pigs at 0, 1, 3, 5, 7, 10, 14, and 21 dpi for viral load detection by qPCR or IFN-*γ* detection by ELISA kit. Anti-p72 (catalog no. 11.PPA.K.3/5; Ingenasa) and -p30 (catalog no. ASFC-5P; IDvet) antibodies were measured using commercial ELISA kit following the manufacturer’s instructions. At 21 dpi, all surviving pigs were euthanized and the tissues and organs (heart, liver, spleen, lung, kidney, submandibular lymph nodes (SLN), mesenteric lymph nodes (MLN), inguinal lymph nodes (ILN), tonsil and bladder) were collected, and then the viral loads in the tissues were quantified by qPCR.

### ELISA for cytokines

The serum samples of the pigs and the cell supernatants of the PAMs infected with ASFV-ΔI7L or ASFV-WT were harvested to measure IFN-*γ* using a porcine IFN-*γ* ELISA kit (catalog no. ELP-IFNg-1; Raybiotech) according to the manufacturer’s instructions.

### Statistical analysis

Statistical analysis was performed using the SPSS 22.0 software. Differences between groups were examined for statistical significance using the Student’s *t* test. Differences between multiple groups of treatments and multiple time points were analyzed for statistical significance using the one-way ANOVA. An unadjusted *P*-value less than 0.05 was considered to be significant.

## Supporting information

S1 Fig The *I7L* gene mutations during ASFV adaptation to HEK293T cells. (A) Comparison of the nucleotide sequence of the *I7L* gene between the ASFV HLJ/2018 strain (ASFV-WT) and the HEK293T cells-adapted ASFV HLJ/2018-P61 (ASFV-P61). (B) Comparison of the *I7L* genes of various cells-adapted ASFV strains.
(TIF)

S2 Fig The biological characteristics of ASFV-ΔI7L. (A) A Schematic diagram of the genome organization of ASFV-ΔI7L. Created with BioRender.com. (B) Generation of ASFV-ΔI7L in primary porcine alveolar macrophages (PAMs). ASFV-ΔI7L was screened by limiting dilution depending on EGFP fluorescence. (C) Identification of ASFV-ΔI7L by PCR assay. ASFV-ΔI7L was identified by PCR targeting the *I7L* (left) and *EGFP* (right) genes. (D) Identification of ASFV-ΔI7L by transmission electron microscopy. The mature virions (white arrows) were also produced in the ASFV-ΔI7L- or ASFV-WT-infected PAMs. (E) Identification of ASFV-ΔI7L by hemadsorption assay. PAMs were infected with ASFV-ΔI7L or ASFV-WT, the "rosettes" of red blood cells (white arrows) and EGFP fluorescence were observed by a fluorescence microscope. (F) The whole genome of ASFV-ΔI7L was analyzed by next-generation sequencing. No undesired mutations were found in the genome of ASFV-ΔI7L, except the expected 309-bp deletion of the *I7L* gene.
(TIF)

S3 Fig The bioinformatic analysis of differentially expressed genes (DEGs). The DGEs in the ASFV-ΔI7L- or ASFV-WT-infected PAMs were subjected to gene ontology (GO) enrichment analysis at 4 (A) and 20 (C) hpi and to Kyoto Encyclopedia of Genes and Genomes (KEGG) enrichment analysis at 4 (B) and 20 (D) hpi.
(TIF)

S4 Fig pI7L suppresses the IFN-γ-triggered JAK-STAT signaling pathway. (A–C) ASFV-ΔI7L induces higher production of IFN-*γ*-stimulated genes (ISGs) than does ASFV-WT. Primary porcine alveolar macrophages (PAMs) were either infected with ASFV-ΔI7L or ASFV-WT or mock-infected at a multiplicity of infection of 1. At 24 hours postinfection, the transcriptional levels of *SOCS1* (A), *GBP1* (B), and *CXCL9* (C) in the cell lysates were quantified by a reverse transcription-quantitative PCR (RT-qPCR). (D) pI7L inhibits the production of ISGs. HEK293T cells were transfected with pFlag-I7L or p3xFlag-CM V-10. At 24 hours posttransfection, the cells were mock-treated or treated with IFN-*γ* (20 ng/ml) for another 12 hours, and then the total RNA was extracted, and the transcriptional level of *SOCS1* in the cell lysates was quantified by RT-qPCR. Error bars denote the standard errors of the means. All the data were analyzed using the one-way ANOVA. ***, *P* < 0.001; ns, not significant.
(TIF)

S1 Table The primers used in this study. (DOCX)
