# Supplementary figures and images for: The I7L protein of African swine fever virus is involved in viral pathogenicity by antagonizing the IFN-γ-triggered JAK-STAT signaling pathway through inhibiting the phosphorylation of STAT1
Source: PLoS Pathog. 2024 Sep 26;20(9):e1012576. doi: 10.1371/journal.ppat.1012576 (PMC11460700; doi:10.1371/journal.ppat.1012576)

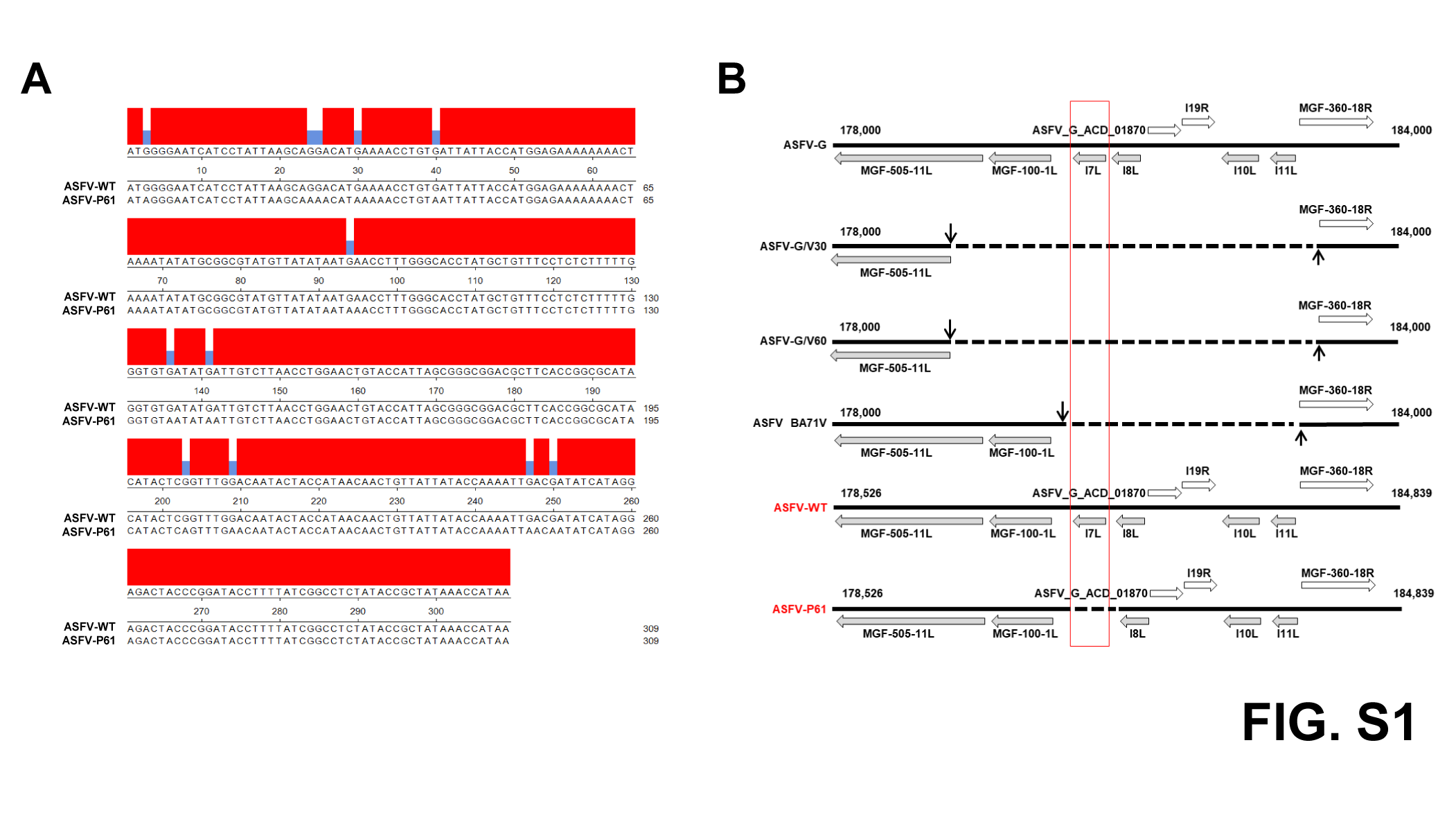

Supplement: S1 Fig — (A) Comparison of the nucleotide sequence of the I7L gene between the ASFV HLJ/2018 strain (ASFV-WT) and the HEK293T cells-adapted ASFV HLJ/2018-P61 (ASFV-P61). (B) Comparison of the I7L genes of various cells-adapted ASFV strains. (TIF) [file ppat.1012576.s001.tif]

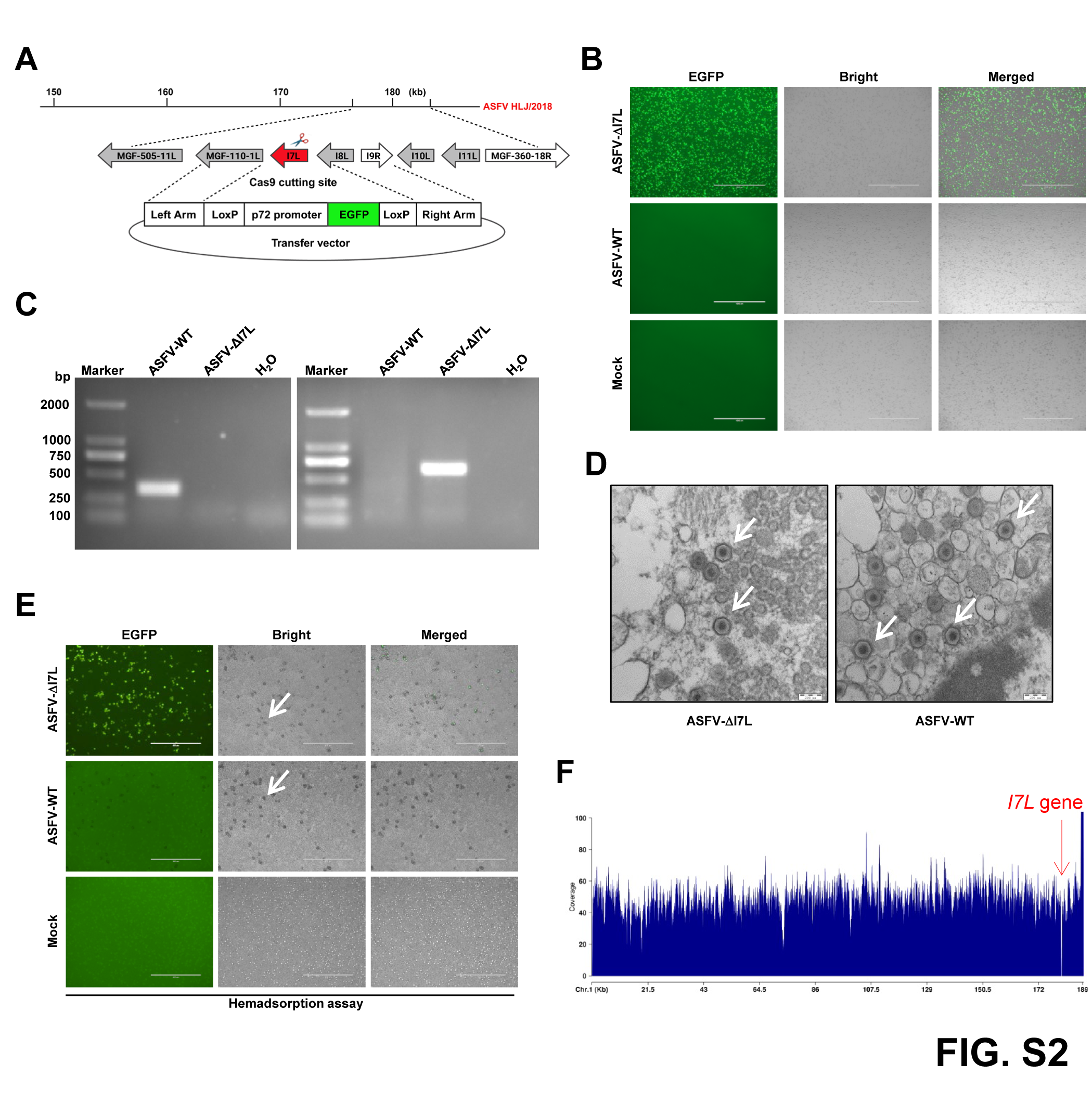

Supplement: S2 Fig — (A) A Schematic diagram of the genome organization of ASFV-ΔI7L. Created with BioRender.com. (B) Generation of ASFV-ΔI7L in primary porcine alveolar macrophages (PAMs). ASFV-ΔI7L was screened by limiting dilution depending on EGFP fluorescence. (C) Identification of ASFV-ΔI7L by PCR assay. ASFV-ΔI7L was identified by PCR targeting the I7L (left) and EGFP (right) genes. (D) Identification of ASFV-ΔI7L by transmission electron microscopy. The mature virions (white arrows) were also produced in the ASFV-ΔI7L- or ASFV-WT-infected PAMs. (E) Identification of ASFV-ΔI7L by hemadsorption assay. PAMs were infected with ASFV-ΔI7L or ASFV-WT, the "rosettes" of red blood cells (white arrows) and EGFP fluorescence were observed by a fluorescence microscope. (F) The whole genome of ASFV-ΔI7L was analyzed by next-generation sequencing. No undesired mutations were found in the genome of ASFV-ΔI7L, except the expected 309-bp deletion of the I7L gene. (TIF) [file ppat.1012576.s002.tif]

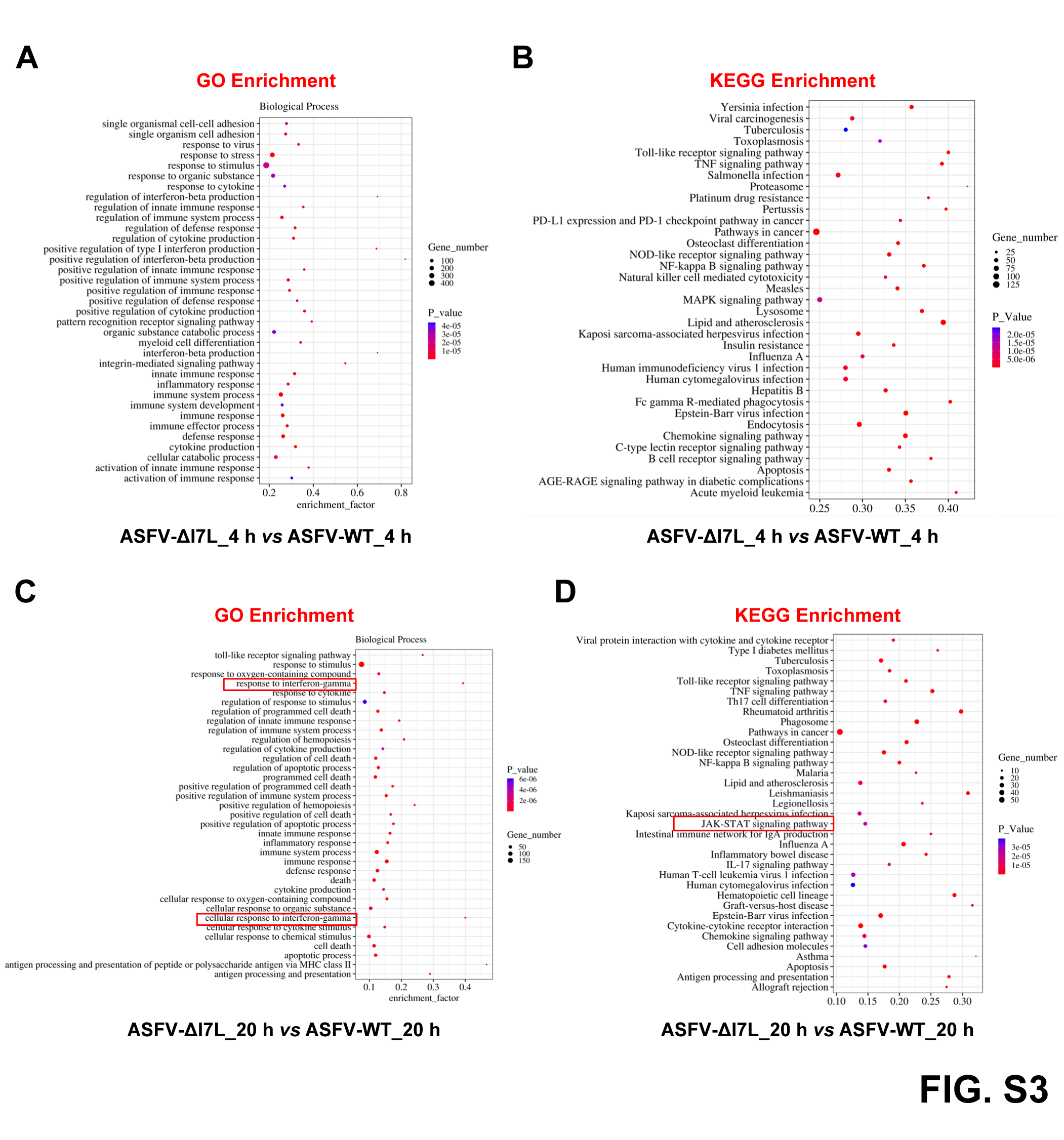

Supplement: S3 Fig — The DGEs in the ASFV-ΔI7L- or ASFV-WT-infected PAMs were subjected to gene ontology (GO) enrichment analysis at 4 (A) and 20 (C) hpi and to Kyoto Encyclopedia of Genes and Genomes (KEGG) enrichment analysis at 4 (B) and 20 (D) hpi. (TIF) [file ppat.1012576.s003.tif]

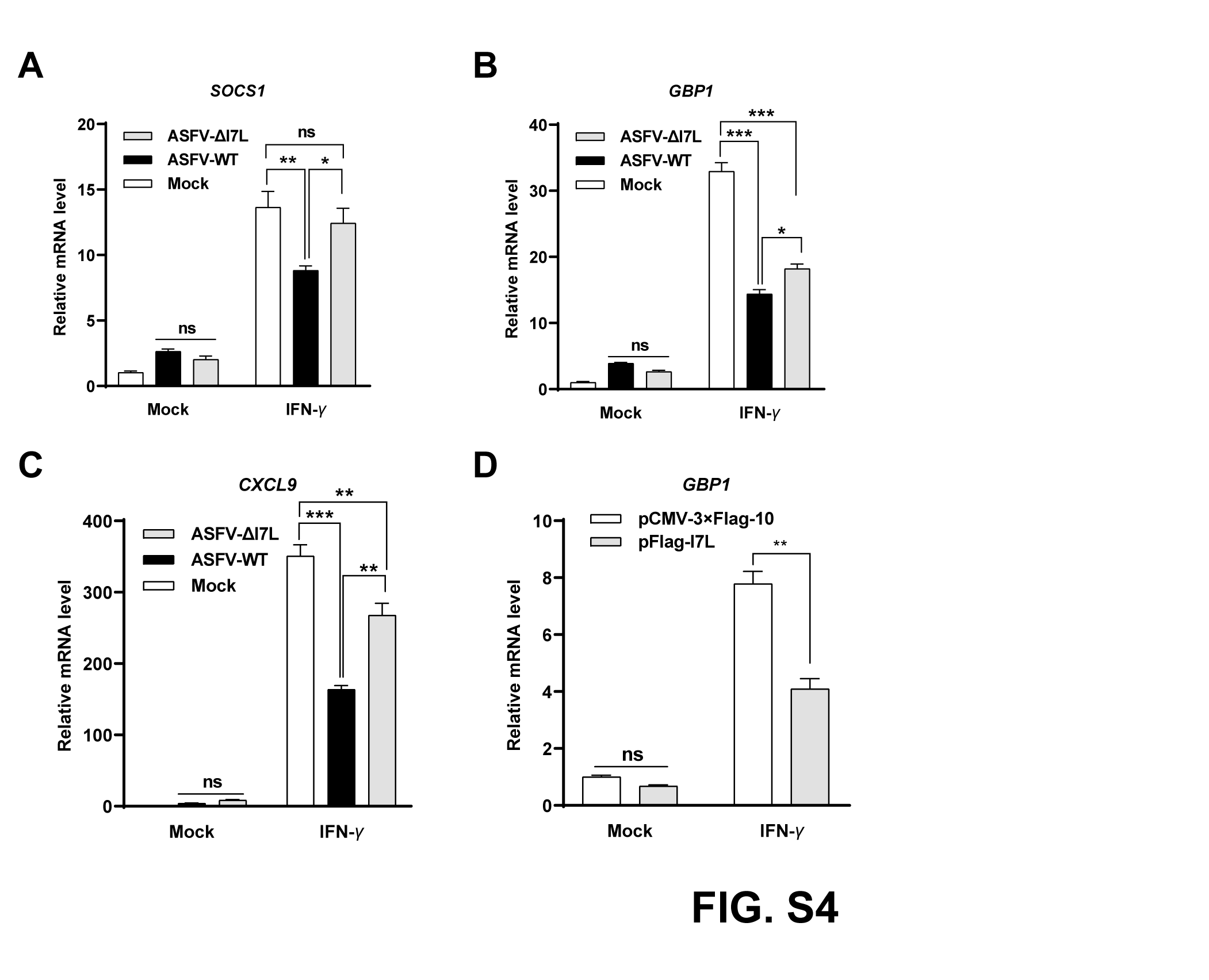

Supplement: S4 Fig — (A–C) ASFV-ΔI7L induces higher production of IFN-γ-stimulated genes (ISGs) than does ASFV-WT. Primary porcine alveolar macrophages (PAMs) were either infected with ASFV-ΔI7L or ASFV-WT or mock-infected at a multiplicity of infection of 1. At 24 hours postinfection, the transcriptional levels of SOCS1 (A), GBP1 (B), and CXCL9 (C) in the cell lysates were quantified by a reverse transcription-quantitative PCR (RT-qPCR). (D) pI7L inhibits the production of ISGs. HEK293T cells were transfected with pFlag-I7L or p3xFlag-CM V-10. At 24 hours posttransfection, the cells were mock-treated or treated with IFN-γ (20 ng/ml) for another 12 hours, and then the total RNA was extracted, and the transcriptional level of SOCS1 in the cell lysates was quantified by RT-qPCR. Error bars denote the standard errors of the means. All the data were analyzed using the one-way ANOVA. ***, P < 0.001; ns, not significant. (TIF) [file ppat.1012576.s004.tif]
