# Supplementary material for: The I7L protein of African swine fever virus is involved in viral pathogenicity by antagonizing the IFN-γ-triggered JAK-STAT signaling pathway through inhibiting the phosphorylation of STAT1
Source: PLoS Pathog. 2024 Sep 26;20(9):e1012576. doi: 10.1371/journal.ppat.1012576 (PMC11460700; doi:10.1371/journal.ppat.1012576)
Supplement: S1 Table — (DOCX) [file ppat.1012576.s005.docx]

**S1 Table.** **Primers used in this study**

| **Primers** | **Sequence（5**′**-3**′**）** | **Description** |
| --- | --- | --- |
| dI7L-LA-F | GGAGCTCGAATTCGAAGCTTATATTATCTATCATAACG | Used to amplify the left arm |
| dI7L-LA-R | CATCTCTCACGAGATCGTGACATAAATGTACAAAATCTT |  |
| 72EGFP-F | AAAAGATTTTGTACATTTATGTCACGATCTCGTGAGAGA | Used to amplify the maker gene |
| 72EGFP-R | TTTATTATATATTATCATACTCCTGTGAGATCATGGCAG |  |
| dI7L-RA-F | AGCTGCCATGATCTCACAGGAGTATGATAATATATAATA | Used to amplify the right arm |
| dI7L-RA-R | TCGACGCGTCTGCAGAAGCTTTGATGAGGTGTCTCTAG |  |
| qI7L-F | CTTACAATCCTCTATGGCTTC | Used in qPCR for *I7L* gene |
| qI7L-R | TCGTCCTTGAAGAAGATGG |  |
| qEGFP-F | AGCAAGAGCGTGTCAATA | Used to identify the recombinant virus |
| qEGFP-R | CGTTGTGGCTGTTGTAGT |  |
| pFlag-I7L-F | CGGAATTCAATGGGGAATCATCCTATTAAG | Used to amplificate the *I7L* gene and  its mutants |
| pFlag-I7L-R | GGGGTACCTTATGGTTTATAGCGGTATAG |  |
| pFlag-I7L(Y94A)-R | GGGGTACCTTATGGTTTATAGCGGTATAGAGGCCGAGCAAAG |  |
| pFlag-I7L(Y98A)-R | GGGGTACCTTATGGTTTATAGCGGGCTAG |  |
| pFlag-I7L(Y100A)-R | GGGGTACCTTATGGTTTAGCGCGGTA |  |
| pFlag-I7L(Y94/100A)-R | GGGGTACCTTATGGTTTAGCGCGGTATAGAGGCCGAGCA |  |
| pGST-I7L-F | CGGGATCCATGGGGAATCATCCTATT |  |
| pGST-I7L-R | CGGAATTCTTATGGTTTATAGCGGTA |  |
| pMyc-IFNGR1-F | CGGAATTCGGATGGCTCTTCTCCTCTTC | Used to amplificate the *IFNGR1* gene  and its mutants |
| pMyc-IFNGR1-R | CCCTCGAGTCAGGAAAACTCCTCGGAT |  |
| pMyc-IFNGR1(ICD)-F | CGGAATTCGGATGAGGAAAATGAATCCA |  |
| pMyc-IFNGR1(ΔTM)-F | TGGATTCATTTTCCTAGAATTTTCTGTGCT |  |
| pMyc-IFNGR1(ΔTM)-R | AGCACAGAAAATTCTAGGAAAATGAATCCA |  |
| pMyc-JAK1-F | CGGAATTCGGATGGCTTTTTGTGCTAAAATG | Used to amplificate the *JAK1* gene |
| pMyc-JAK1-R | CCCTCGAGTTATTTTAAAAGTGCTTCAAATCC |  |
| pMyc-JAK2-F | CGGAATTCGGATGGGAATGGCTTGCCTC | Used to amplificate the *JAK2* gene |
| pMyc-JAK2-R | CCCTCGAGTCAAGCCATACTGTCCCTT |  |
| pMyc-STAT1-F | CGGAATTCGGATGTCCCAGTGGTATGAGC | Used to amplificate the *STAT1* gene  and its mutants |
| pMyc-STAT1-R | CCCTCGAGTTAGTCAAGGTTCATAGTTC |  |
| pMyc-STAT1(Y701A)-F | CCCTAAAGGAACAGGAGCCATCAAGACTGAATTGA |  |
| pMyc-STAT1(Y701A)-R | TCAATTCAGTCTTGATGGCTCCTGTTCCTTTAGGG |  |
| qhGAPDH-F | GACACCCACTCCTCCACCTTT | Used in qPCR for human *gapdh* gene |
| qhGAPDH-R | ACCACCCTGTTGCTGTAGCC |  |
| qhIRF1-F | GAGGAGGTGAAAGACCAGAGCA | Used in qPCR for human *irf1* gene |
| qhIRF1-R | TAGCATCTCGGCTGGACTTCGA |  |
| qhCXCL10-F | GGTGAGAAGAGATGTCTGAATCC | Used in qPCR for human *cxcl10* gene |
| qhCXCL10-R | GTCCATCCTTGGAAGCACTGCA |  |
| qhGBP1-F | TAGCAGACTTCTGTTCCTACATCT | Used in qPCR for human *gbp1* gene |
| qhGBP1-R | CCACTGCTGATGGCATTGACGT |  |
| qpGAPDH-F | GAAGGTCGGAGTGAACGGATTT | Used in qPCR for pig *gapdh* gene |
| qpGAPDH-R | TGGGTGGAATCATACTGGAACA |  |
| qpGBP1-F | GAAGGGTGACAACCAGAACGAC | Used in qPCR pig for *gbp1* gene |
| qpGBP1-R | AGGTTCCGACTTTGCCCTGATT |  |
| qpSOCS1-F | CTGCTTCTTCGCCCTCAGTGTG | Used in qPCR for pig *socs1* gene |
| qpSOCS1-R | CCAGCAGCTCGAAGAGGCAGTC |  |
| qpIFN-*γ*-F | ACCTAATGGTGGACCTCTT | Used in qPCR for pig *ifng* gene |
| qpIFN-*γ*-R | GCCTTGGAACATAGTCTGA |  |
| qpIRF1-F | CCTGATACCTTCTCTGATGG | Used in qPCR for pig *irf1* gene |
| qpIRF1-R | CAACTTCTGGCTCTTCCTT |  |
| qpCXCL9-F | CATCTTCCTGACTCTGATTG | Used in qPCR for pig *cxcl9* gene |
| qpCXCL9-R | ACCTGTTTCTCCCACTCT |  |
| qpCXCL10-F | CTGCCTTATTCTTCTGACTCT | Used in qPCR for pig *cxcl10* gene |
| qpCXCL10-R | CCTCGGATTAACAGGTCTG |  |
| (LA) left arm; (RA) right arm; (d) deletion; (q) quantitative ; (F) forward; (R) reverse; (h) Human; (p) pig. | | |
